# The DNA-Binding Domain of *S*. *pombe* Mrc1 (Claspin) Acts to Enhance Stalling at Replication Barriers

**DOI:** 10.1371/journal.pone.0132595

**Published:** 2015-07-22

**Authors:** Juergen Zech, Emma Louise Godfrey, Hisao Masai, Edgar Hartsuiker, Jacob Zeuthen Dalgaard

**Affiliations:** 1 Warwick Medical School, University of Warwick, Gibbet Hill Campus, CV47AL Coventry, United Kingdom; 2 Genome Dynamics Project, Department of Genome Medicine, Tokyo Metropolitan Institute of Medical Science, Setagaya-ku, Tokyo 156–8613, Japan; 3 School of Biological Sciences, Bangor University, Deiniol Road, Bangor, Wales, LI57 2UW, United Kingdom; University College London, UNITED KINGDOM

## Abstract

During S-phase replication forks can stall at specific genetic loci. At some loci, the stalling events depend on the replisome components *Schizosaccharomyces pombe* Swi1 (*Saccharomyces cerevisiae* Tof1) and Swi3 (*S*. *cerevisiae* Csm3) as well as factors that bind DNA in a site-specific manner. Using a new genetic screen we identified Mrc1 (*S*. *cerevisiae* Mrc1/*metazoan* Claspin) as a replisome component involved in replication stalling. Mrc1 is known to form a sub-complex with Swi1 and Swi3 within the replisome and is required for the intra-S phase checkpoint activation. This discovery is surprising as several studies show that *S*. *cerevisiae* Mrc1 is not required for replication barrier activity. In contrast, we show that deletion of *S*. *pombe mrc1* leads to an approximately three-fold reduction in barrier activity at several barriers and that Mrc1’s role in replication fork stalling is independent of its role in checkpoint activation. Instead, *S*. *pombe* Mrc1 mediated fork stalling requires the presence of a functional copy of its phylogenetically conserved DNA binding domain. Interestingly, this domain is on the sequence level absent from *S*. *cerevisiae* Mrc1. Our study indicates that direct interactions between the eukaryotic replisome and the DNA are important for site-specific replication stalling.

## Introduction

The process of genome duplication is a significant challenge to the cell as genetic and epi-genetic information have to be precisely copied as well as the genome integrity maintained. Interestingly, the replication forks do not progress along the DNA at an uniform rate and can pause or terminate at so called site-specific replication barriers (reviewed in Ref. [1]). Site-specific barriers exist in two main types; “DNA-binding protein” mediated and “hard to replicate” sequences. The latter being repetitive DNA sequences that can form noncanonical stable secondary structures such as hairpins, cruciforms, triplexes and quadruplexes [2–11]. Protein-mediated barriers generally are thought to act to maintain genomic stability by preventing the collision of the transcription machinery with replication forks and the subsequent formation of dysfunctional replication forks (reviewed in Ref. [1]). However, the process of stalling replication at such barriers can itself lead to DNA instability [12,13] and in fission yeast replication barriers have been shown to mediate a program of cellular differentiation involving DNA rearrangements (see below; reviewed in Ref. [14]).

Protein-mediated replication-stalling events are generally mediated by two types of *trans*-acting factors. One type moves with the replication fork, while the other type consists of DNA binding proteins, which are statically bound to site-specific *cis*-acting DNA elements at the barrier loci. *S*. *pombe* Swi1 (*S*. *cerevisiae* Tof1) and Swi3 (*S*. *cerevisiae* Csm3) are factors of the first type [15–21]. In both *S*. *cerevisiae* and *S*. *pombe*, these two factors have been shown to travel with the replication fork and in *S*. *cerevisiae* Tof1 and Csm3 have been shown to be integral parts of the replisome [18–21]. Similarly, the human homologues TIMELESS (Swi1) and TIPIN (Swi3) interact to form a complex, and co-localize with PCNA [22]. Swi1/Tof1 and Swi3/Csm3 mediate stalling of the replication forks at loci where the second type of static barrier proteins are bound. Recently this has been shown also for TIMELESS at the human *rDNA* barrier [23]. The best studied loci include binding sites of Sap1, Reb1, and Rtf1 in *S*. *pombe* as well as Fob1 and kinetochores in *S*. *cerevisiae* [24–29]. Importantly, in the absence of Swi1/Tof1 and Swi3/Csm3 there is a complete loss of barrier activity at these genetic loci [24–29]. Swi1/Tof1 has a more complex role at stalled forks at *tRNA* genes and at sequences that can form stable DNA secondary structures [2,29,30].

Swi1 and Swi3 possess functional activities connected with the control of S-phase progression in addition to their replication barrier activity. Swi1 and Swi3 as well as their *S*. *cerevisiae* homologues form a trimeric complex with the S-phase checkpoint mediator Mrc1 [31,32]. In *S*. *cerevisiae* this complex of Mrc1, Tof1 (Swi1) and Csm3 (Swi3) can be co-purified with other known replisome components [19,31]. Moreover, Swi1, Swi3, and Mrc1 also act in the checkpoint response, activated by replication stress, from the “sensor kinase” Rad3 (*S*. *cerevisiae* Mec1/ Metazoan ATR) to the effector kinase Cds1 (*S*. *cerevisiae* Rad53/ Metazoan Chk1) [33–35]. However, it is important to highlight that deletion experiments of *mrc1*, *swi1*(*tof1*) and *swi3*(*csm3*) indicate functional differences between the genes. For example, Cds1 phosphorylation observed in cells treated with HU is completely lost in an *mrc1* deletion background, while only a reduction in Cds1 phosphorylation is observed when *swi1* or *swi3* are deleted [20,35,36]. Similarly, while a deletion of *tof1*, the *S*. *cerevisiae* homologue of Swi1, only has a minor effect on the general rate of S-phase progression, deletion of *S*. *cerevisiae mrc1* leads to a significant reduction [29,37,38]. Also, while *S*. *cerevisiae* Tof1 is required for replication protein-mediated barrier activity at the Fob1 barrier in the *rDNA*, and at several *tRNA* genes and centromeres investigated, a deletion of *S*. *cerevisiae* Mrc1 does not affect stalling at these loci [17,29,38,39].

In summary the molecular mechanism that underlies replication-stalling events at natural barriers is not well understood to date and its study is complicated by the fact that several important factors seem to be active in multiple pathways. However, the following recent discoveries have improved our understanding of which factors are involved in replication fork stalling as well as the roles they are playing. Using a new screening tool based on the mating-type switching system of *S*. *pombe* the flavine adenine dinucleotide-dependent lysine-specific demethylase enzymes, Lsd1 and Lsd2, were identified as required for replication stalling at several replication barriers [40]. These barriers included the *MPS1* and *RTS1* elements in the mating-type region as well as the *rDNA* barrier element. It is not known how Lsd1 and Lsd2 act at these elements, but experiments suggest that both enzymes have structural and catalytic roles in mediating replication fork stalling. It has also been shown in *S*. *cerevisiae* that Tof1 (*S*. *pombe* Swi1) and Csm3 (*S*. *pombe* Swi3) counteract the helicase Rrm3 to mediate replication barrier activity [17]. Rrm3 is a helicase that travels with the replication fork and is required for the efficient removal of non-histone proteins in front of the fork [41]. Furthermore, an amino-acid substitution has been identified in Swi1 that abolishes barrier activity of Rtf1 at the *RTS1* element but that does not affect barrier activity of other barriers investigated [15]. This suggests that specific protein-protein interactions between Rtf1 and Swi1 might be important for replication stalling at the *RTS1* element. Finally, the *S*. *pombe* factor Rtf2 has been shown to act to prevent Srs2 mediated replication restart, thus promoting termination, at the *RTS1* element [42].

**Fig 1:**
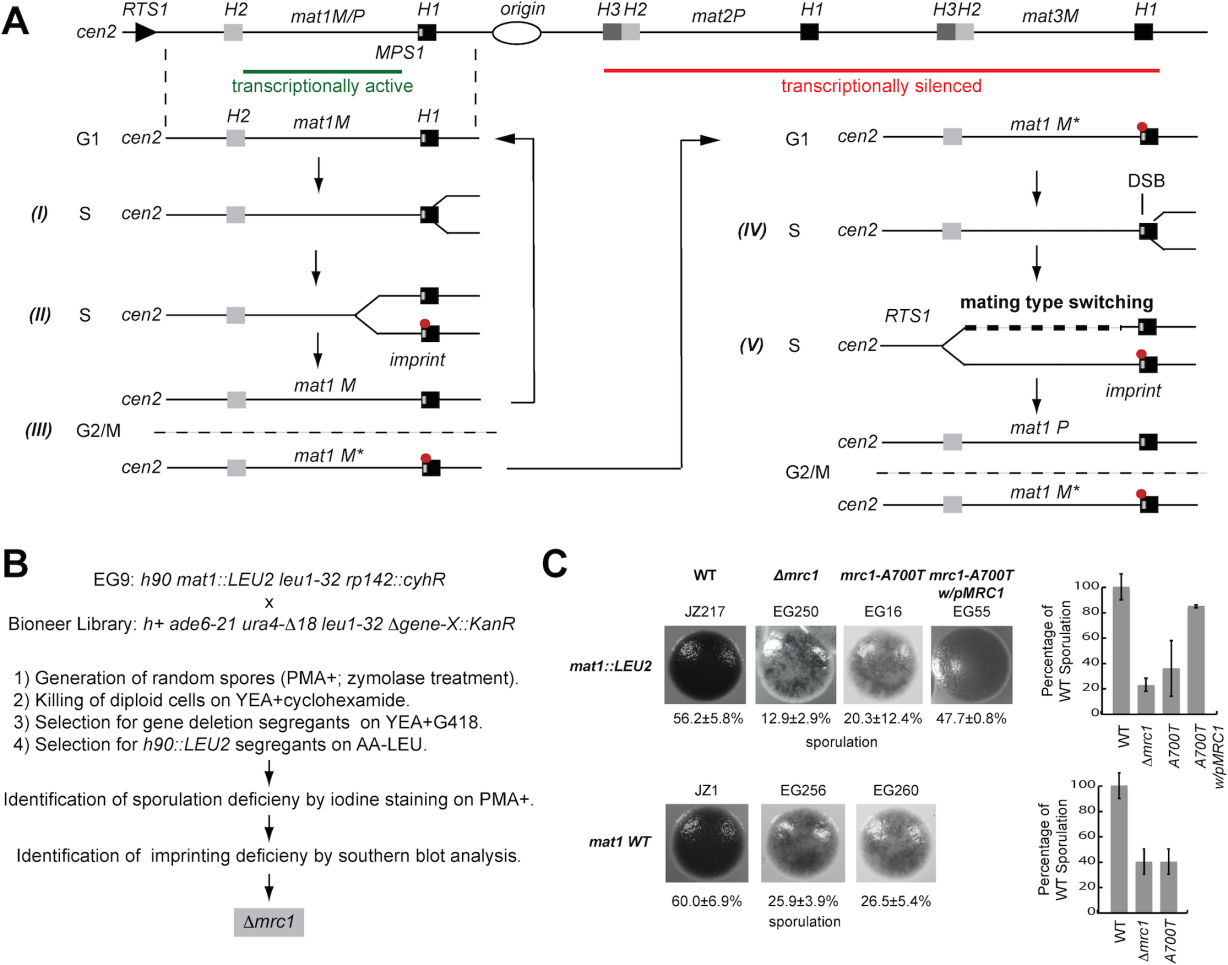
The use of mating-type switching of *S*. *pombe* as a screening tool to identify genes, influencing the stalling of DNA replication forks at the *MPS1* site. (A) Mechanism of mating-type switching in *S*. *pombe*: *S*. *pombe* cells can switch between two different mating-types *Plus* (*P*) and *Minus* (*M*). Top line drawing. The mating-type of a cell is determined by the *mat1* locus, which can contain either *P* or *M* information. Switching involves the precise replacement of the mating-type cassette at *mat1* with the opposite mating-type information through a recombination event that utilizes one of two donor-loci, located centromere-distal to *mat1*, *mat2P* or *mat3M*, as donors of the genetic information (top line-drawing). In addition, cells of the two mating-types can either be un-switchable (M, P) or switchable (M*, P*); switchable cells carry a ribonucleotide imprint at the *mat1* locus (see below). Importantly, the *mat1* locus is replicated in a uni-directional manner due to the presence of a terminator of replication (the *RTS1* element) on the centromere-proximal (cen) side. Lower line drawings. (I) When DNA replication takes place in S-phase, the replisome replicating *mat1* pauses at the *MPS1* barrier located at the boundary of the *mat1* cassette. This pause leads to the site-specific priming of an Okazaki fragment. (II) The replication fork then progresses on, and the primer from the Okazaki fragment is converted into an imprint consisting of two ribonucleotides incorporated into the DNA. (III) After cell division, this imprint is inherited by one daughter cell (M*) making it capable of switching mating type: (IV) In the following S-phase a break is introduced at the site of the imprint, when the leading-strand runs into the imprint present in the template strand, (V) leading to the induction of the recombination event (bold dashed line) that underlies mating-type switching. (B) To identify factors involved in replication pausing at the *MPS1* site, the Bioneer knockout library was crossed with an *h* ^*90*^ strain that had been tagged at the *mat1* locus with a *S*. *cerevisiae LEU2* genetic marker. The sporulation phenotype of different gene knockouts was examined after selection on YEA+G418+cyclohexamide (the different genes are knocked out with a *Kan* ^*R*^ cassette, and cyclohexamide kills diploid cells due the recessive *cyh* ^*r*^ mutation) followed by selection on AA-Leu (*LEU2 is* linked with *mat1*). Low and non-sporulating strains were identified by iodine staining of strains grown on sporulation media (PMA+), and these candidate strains where analysed by Southern blot analysis to further assess the level of *mat1* imprinting. *Δmrc1* was identified and verified as a candidate that influenced pausing at the *MPS1* barrier. (C) Sporulation staining phenotype and sporulation levels of *Δmrc1* generated from the Bioneer knockout library, as well as of the *mrc1-A700T* (K234Stop) generated according to Holmes, et al. [40]. The low sporulation phenotype of the *Δmrc1* stain can be complemented by the transformation of a plasmid containing a genomic copy of the *mrc1* gene (pMRC1). Strain names are given above each panel, and the percentage of spores observed in the colonies by confocal microscopy is given below. Graphs to the right display the level of sporulation observed in the mutant colonies relative to wild-type colonies (100%).

In this study, we utilize the mating-type switching system in a novel genetic screen to identify *S*. *pombe* Mrc1 (*metazoan* Claspin) as required for efficient replication stalling (Fig 1A). We show that *S*. *pombe* Mrc1 has a general role mediating efficient stalling at several replication barriers, including *MPS1*, *RTS1*, the *rDNA* barrier and a *tRNA* gene. This novel function of *S*. *pombe* Mrc1 is independent of the protein’s checkpoint activity, but dependent on a helix-turn-helix DNA-binding domain. This domain has been shown to bind DNA in a non-site specific way with a preference for branched DNA structures [43]. Importantly, this DNA-binding domain is phylogenetically conserved in a wide range of eukaryotic *mrc1* (CLASPIN) genes except in *S*. *cerevisiae mrc1*. This suggests that there might be specific differences between the mechanism of replication stalling in *S*. *cerevisiae* and *S*. *pombe*.

## Results

### Using mating-type switching as a tool to identify potential factors required for fork stalling

The mating-type switching system of fission yeast *S*. *pombe* is an excellent model system to study replication fork stalling, because it is dependent on a replication-coupled recombination event that is established through the involvement of several replication barriers ([15]; Fig 1A) and produces an easily detectable phenotype. The high sporulation levels in colonies of wild-type *h* ^*90*^ strains result from a high mating-type switching rate. This strains display a dark staining phenotype when exposed to iodine vapours due to the presence of starch compounds in the spores [44]. Genetic alterations or mutations that lead to a reduced rate of mating-type switching (e.g. by affecting the replication-coupled recombination event by changing replication barrier activity) result in a skewed ratio of M and P cells. The consequences are less frequent mating and a decrease in sporulation levels causing an easily detectable low or speckled staining phenotype.

The replication-coupled recombination event includes the following steps (Fig 1A). Pausing of the replication fork at the *MPS1* element located at the mating-type locus *mat1* is required for the introduction of an imprint that consists of two ribonucleotides incorporated into the DNA [45–47]. Experiments suggest that these ribonucleotides originate from the primer of an Okazaki fragment that is laid down in response to the replication pause [48]. This ribonucleotide imprint is maintained in the DNA throughout one generation, and during the next S-phase acts as a barrier for leading-strand replication, inducing a recombination event that leads to mating-type switching (reviewed in Ref. [14]). In addition to *MPS1* and the imprint, a replication barrier named *RTS1* is present in the mating-type region [49]. *RTS1* acts to optimize mating-type switching by ensuring uni-directional replication at the *mat1* locus. The activity of both *MPS1* and *RTS1* depend on Swi1 and Swi3 [26,48].

Consequently, mutations that reduce replication pausing at the MPS1 site cause a sporulation deficient phenotype [15,44]. Therefore, we crossed the non-switchable version 2 *S*. *pombe* Bioneer gene deletion library with a strain wild-type for mating-type switching (Fig 1B). The latter strain had been tagged with a *S*. *cerevisiae LEU2* gene in the mating-type locus, allowing us to create a library of genetic segregants that carried both the gene deletions (G418 resistance) and the *h* ^*90*^ wild-type mating-type locus (leucine prototrophs). Colonies obtained from these deletion strains were stained with iodine vapour, to evaluate the effect of the individual deletions on sporulation. More than 425 deletions affected the efficiency of sporulation. Among these 178 lead to a severe or complete loss of sporulation including several genes already known to be required for efficient mating-type switching, such as *swi3* (see S2 Table). This demonstrated the capability of our screening method to identify genes involved in replication fork pausing. It should be noted that a *swi1* deletion strain is not present in this Bioneer library.

We identified the *mrc1* deletion as our best candidate for being a mutation affecting replication pausing. Firstly, several studies have previously shown that Mrc1 is a component of the replisome in both *S*. *pombe* and *S*. *cerevisiae* [19,31,32]. Furthermore, independent from our original screen we identified a mrc1 nonsense mutation (*mrc1-A700T*), in a screen recently described by Holmes et al. that specifically identifies mutants that affect pausing and imprinting [40].

**Fig 2:**
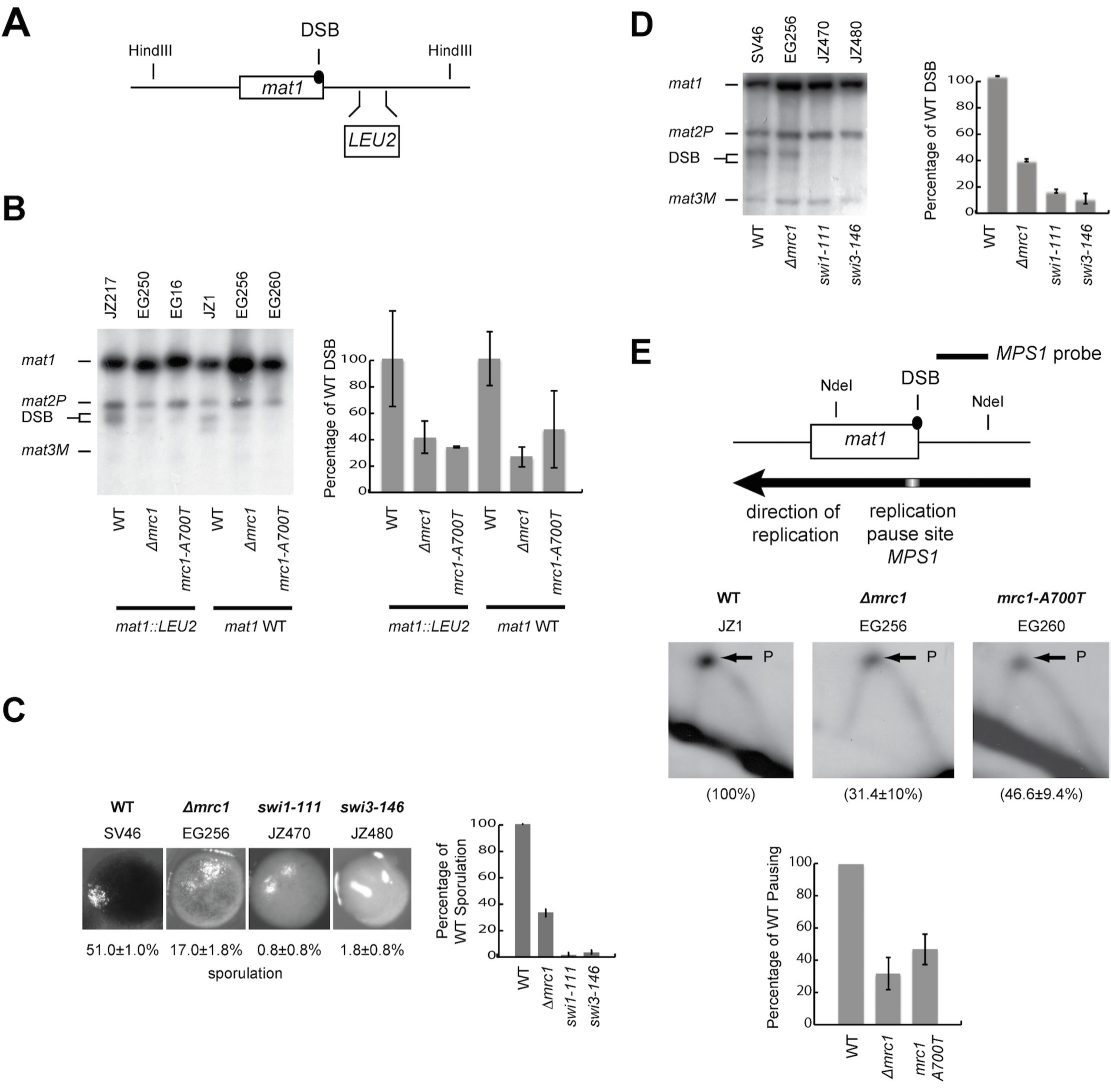
Characterization of the *mrc1* mutations. (A) Top line-drawing; schematic representation of the mating type region showing the 10.4 kb *Hin*dIII fragment containing the *mat1* locus. The positions of the imprint-dependent DSB and of the *S*. *cerevisiae LEU2* gene inserted in the *mat1* region (strain JZ217) are indicated. (B) Left panel, Southern blot of *Hind*III-digested DNA probed with a *mat1P* specific probe. This probe hybridises to the *mat1* (10.4 kb), *mat2P* (6.3 kb) and *mat3M* (4.2 kb) cassettes as well as to the *mat1* DSB products (5.0 and 5.4 kb, DSB). Above the panel, strains names are given. Below the panel the *mrc1* alleles (vertical bold text) and the *mat1* alleles (horizontal text) are given. To the right a graph displays the mean level of DSBs in the strains relative to the wild-type level (100%). The values are based on two measurements, with values indicated with vertical lines. (C) Comparison of sporulation levels of the *Δmrc1* strain to *swi1-111* and *swi3-146* strains. To the right a graph displays the mean level of sporulation in the strains relative to the wild-type level (100%). See Fig 1C for description. (D) Comparison of imprinting levels between *Δmrc1*, *swi1-111* and *swi3-146* strains. For description see panel 1B. (E) Top line-drawing; WT; schematic representation of the 2.7 kb NdeI fragment of the mating-type region used to examine replication fork pausing at the *mat1 MPS1*. The position of the DSB, the polarity of replication in this region (black arrow), the replication pause site *MPS1* and the position of the probe used to hybridise 2D-gels of this region are shown. Middle panels, quantification of replication pausing in wild type and *mrc1* strains at the *MPS1* site (the pause signal is indicated with an arrow and P). Genotypes and strain names are given above the 2D-gel panels. Below the panels the intensity of the replication fork pause signal is shown for each strain as a percentage of the WT pause signal’s intensity. Two independent experiments were performed and the mean is given. Lower panel, graph displaying the data obtained above.

This study focuses on the further characterization of the function of the *mrc1* gene. First, we quantified the effect the identified *mrc1* mutations have on sporulation efficiency and confirmed the genetic correlation between the *mrc1* mutants and the sporulation deficient phenotype. The *Δmrc1* mutant strain displayed 12.9% sporulation corresponding to 23% of the wild type levels (Fig 1C; EG250). A slightly greater reduction was observed when an allele of the wild-type mating-type region (lacking the *LEU2* gene) was combined with the *Δmrc1* allele (Fig 1C; EG256). An experimental comparison of the sporulation of the *mrc1* deletion strain to functional null mutations in the *swi1* and *swi3* genes, showed that while the *swi1* and *swi3* mutations almost abolish sporulation, the *mrc1* mutation only leads to a 3–4 fold reduction in sporulation (Fig 2C). Backcrossing experiments using the mutant *mrc1* strain to the parental wild type strain did not detect any crossovers between the low-switching phenotype and the Kan^r^ marker gene used to delete the *mrc1* gene in the 22 tetrads analysed. In addition, the nonsense mutation in *mrc1A700T* displayed 26.5% sporulation corresponding to 44% of the wild-type levels (Fig 1C; EG260). Finally, the low-sporulation phenotype of the *mrc1-A700T* mutant could be complemented by a plasmid containing the wild-type genomic allele of *mrc1* (Fig 1C; EG55). In summary, these data show that loss of Mrc1 function is correlated in *S*. *pombe* with a reduction of the ability to sporulate.

### Mrc1 is required for efficient replication stalling at *MPS1*

To determine whether the reduction of sporulation resulting from the *Δmrc1* mutation was due to defects in imprinting and replication pausing, the genomic DNA from mutant and control strains was purified using the method described by Dalgaard and Klar [45]. Using this method the imprint at *mat1* is efficiently converted into a double strand break (DSB). The analysis of wild type, *Δmrc1* and *mrc1-A700T* strains showed that, while the imprint was easily detectable in the wild-type strain, it was significantly reduced in the *Δmrc1* (EG250) and *mrc1-A700T* (EG16) strains to 40.8% and 33.8% of the wild-type level, respectively (Fig 2A & 2B). A comparison to the *swi1-111* and *swi3-146* strains showed that while loss of Swi1 and Swi3 function abolishes imprinting, the *mrc1* deletion only leads to a reduction in imprinting (Fig 2D).

We then tested whether the loss of Mrc1 affected pausing of the replication fork at *MPS1*. Comparison of the pausing signal in wild type and *Δmrc1* replication intermediates showed that there was an approximately three-fold reduction in the mutant background (Fig 2E). This is a similar fold reduction to that observed for the level of sporulation and imprinting as seen in Fig 1C. Therefore, we concluded that Mrc1 is required for efficient pausing at the *MPS1* barrier. However, as in the case of sporulation efficiency and imprinting, the effect of the *mrc1* deletion on pausing was significantly less than that previously observed for the *swi1* and *swi3* deletions, which lead to a complete loss of the *MPS1* barrier signal [15].

### Mrc1 is important for fork stalling at several replication barriers

Mrc1 has been shown to form a complex with Swi1 (*S*. *cerevisiae* Tof1) and Swi3 (*S*. *cerevisiae* Csm3) in both budding and fission yeast [31,50,51]. As mentioned above, the replication proteins Swi1 and Swi3 are required for replication barrier activity at several other genetic *loci* (see above). Therefore, we tested whether Mrc1 also influences the activity at other barriers.

**Fig 3:**
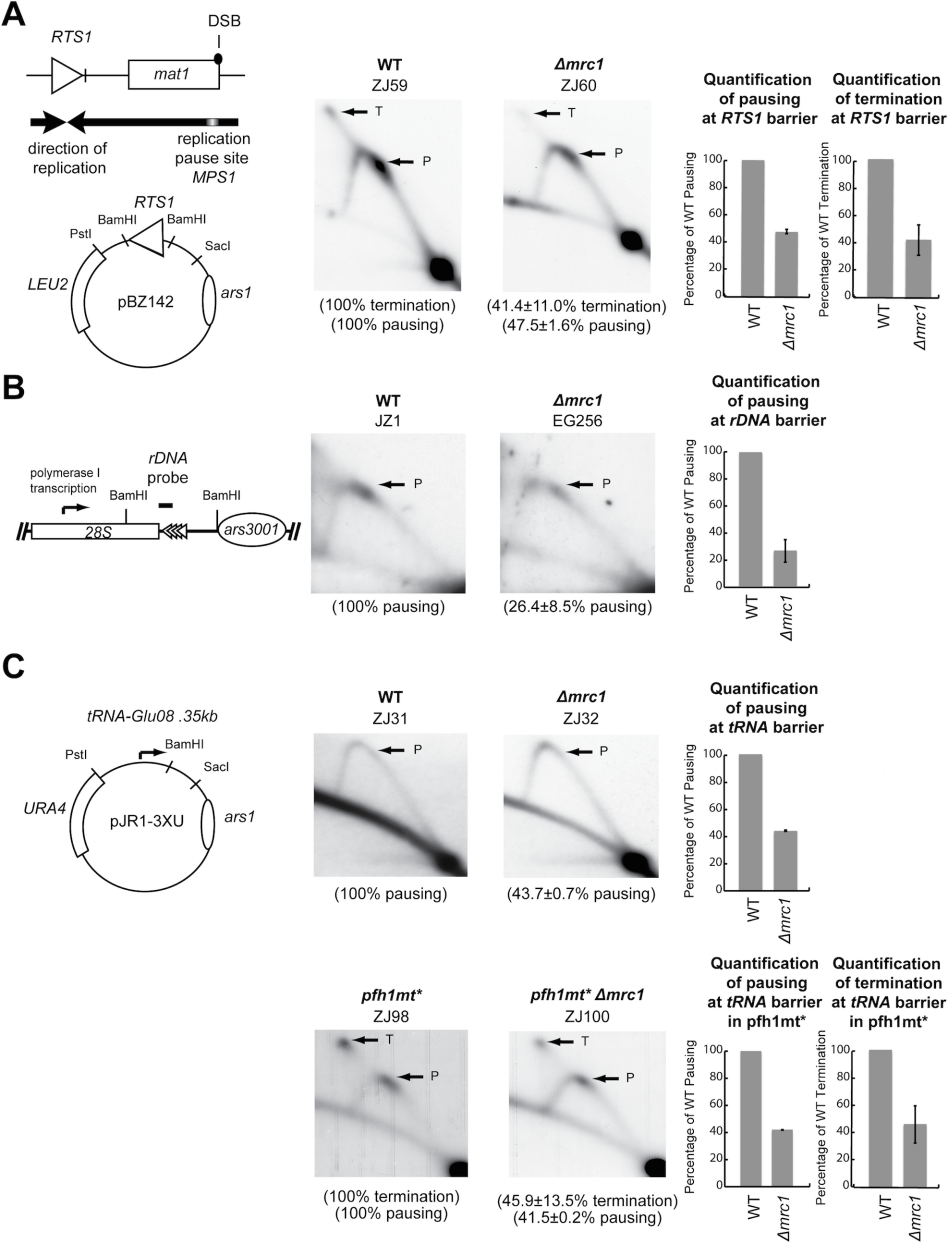
Quantification of the effect the *Δmrc1* mutation has on the replication pausing and termination at different barriers. (A) Left panel, schematic representation of the mating-type region containing the replication termination site *RTS1*, the *mat1* locus, the polarity of replication within the region and the replication pause site *MPS1*. The plasmid pBZ142 contains the *RTS1* site element and the *ars1* origin [83]. Middle and right panel, deletion of *mrc1* reduces replication termination (indicated with an arrow and T) and pausing (indicated with an arrow and P) at the *RTS1* (probed with a 0.8 kb BamHI fragment from pBZ142). Genotypes and strain names are given above the 2D-gel panels, and the relative intensity of the barrier signals below. Graphs; the replication fork pausing and termination signals’ intensities are given for each strain as a percentage of the WT signals. The results given are the mean from two independent experiments. (B) Left panel, schematic representation of the *BamH*I fragment of an *rDNA* repeat containing the gene for 28S ribosomal RNA, the polarity of PolI transcription, the position of the 0.55 kb probe used for the Southern analysis of 2D-gels, the rDNA barriers and the *ars3001* origin [84]. Middle and right panels, see above for description. (C) Left panel, schematic representation of the plasmid pJR-3XU containing the tRNA-Glu08 gene and the *ars1* origin [54]. Middle and right panels, see above for description (probe 0.6 kb BamHI, SacI fragment from pJR1-3XU).

The *RTS1* barrier plays a role in optimising mating-type switching by controlling the direction in which the *mat1* locus is replicated [49]. Quantification of the pause and termination signals at *RTS1* showed that a deletion of *mrc1* reduces both types of barrier signals to 47.5% and 41.4% of the wild-type levels (Fig 3A). To address whether Mrc1 has a role outside the mating-type region we looked at the rDNA replication barrier. This barrier element is located at the 3’-end of the polymerase I transcription unit [16,52]. While we were unable to resolve the sub-barrier elements present at this locus [16,53], our data established that the overall level of barrier signals are reduced to approximately 26.4% of wild-type level in *Δmrc1 strains* (Fig 3B). Finally, we looked at barrier activity at a plasmid-borne *tRNA* gene. This replication barrier has previously been shown to be very weak, only clearly visible in a pfh1-mt* (*S*. *cerevisiae rrm3* and *pif1*, *metazoan PIF1*) mutant background [54]. However, by careful comparison of the very weak barrier signal in the wild-type strain with the corresponding position on the Y-arc in the *Δmrc1* strain using a phosporimager we could measure a reproducible reduction in barrier activity (Fig 3C upper panels). This is supported by the clearly visible reducing effect the *Δmrc1* mutation has on the *tRNA* barrier signal enhanced by the pfh1-mt* mutation. Here a ~60% reduction in intensity is observed for the barrier signal (Fig 3C lower panels). Thus, our data show that Mrc1, like Swi1 and Swi3, is required for replication barrier activity not only at *MPS1* but also at at least three other *S*. *pombe* DNA replication barriers. However, while *swi1* and *swi3* functional-null mutations abolish barrier activity the *mrc1* deletion mutation only leads to a reduction in barrier activity. Finally, we would like to point out that the interaction of the replisome with *tRNA* barriers might be more complex than with other protein mediated barriers. Earlier observations have already shown that *tRNA* barriers behave differently in the absence of Swi1/Tof1 and Swi3/Csm3 than other barriers [24–30] (see introduction). While our findings clearly show a reduction of *tRNA* barrier activity in a Δmrc1 pfh1-mt* strain compared with a pfh1-mt* strain where retained polymerase III complexes form a strong barrier, the apex of the Y-arc of the tRNA barrier appears slightly more intense in the *Δmrc1* background than in the presence of Mrc1 in a strain with wild-type *pfh1*. This could be an indication that Mrc1’s role at tRNA barriers might be more complex and depends on the presence or absence of Pfh1 (e.g. preventing fork stalling at tRNA barriers and ensuring a smooth passage of the replication fork in the presence of Pfh1 versus enhancing fork stalling at tRNA barriers in its presence). However, the signals in the 2D gels in the upper panels of Fig 3C are not strong enough to draw conclusions beyond the fact that *Δmrc1* and not pfh1-mt* causes the reduction in *tRNA* barrier activity observed in the lower panels of Fig 3C.

### Mrc1 is required for fork stalling at *MPS1* in imprinted and unimprinted cells

**Fig 4:**
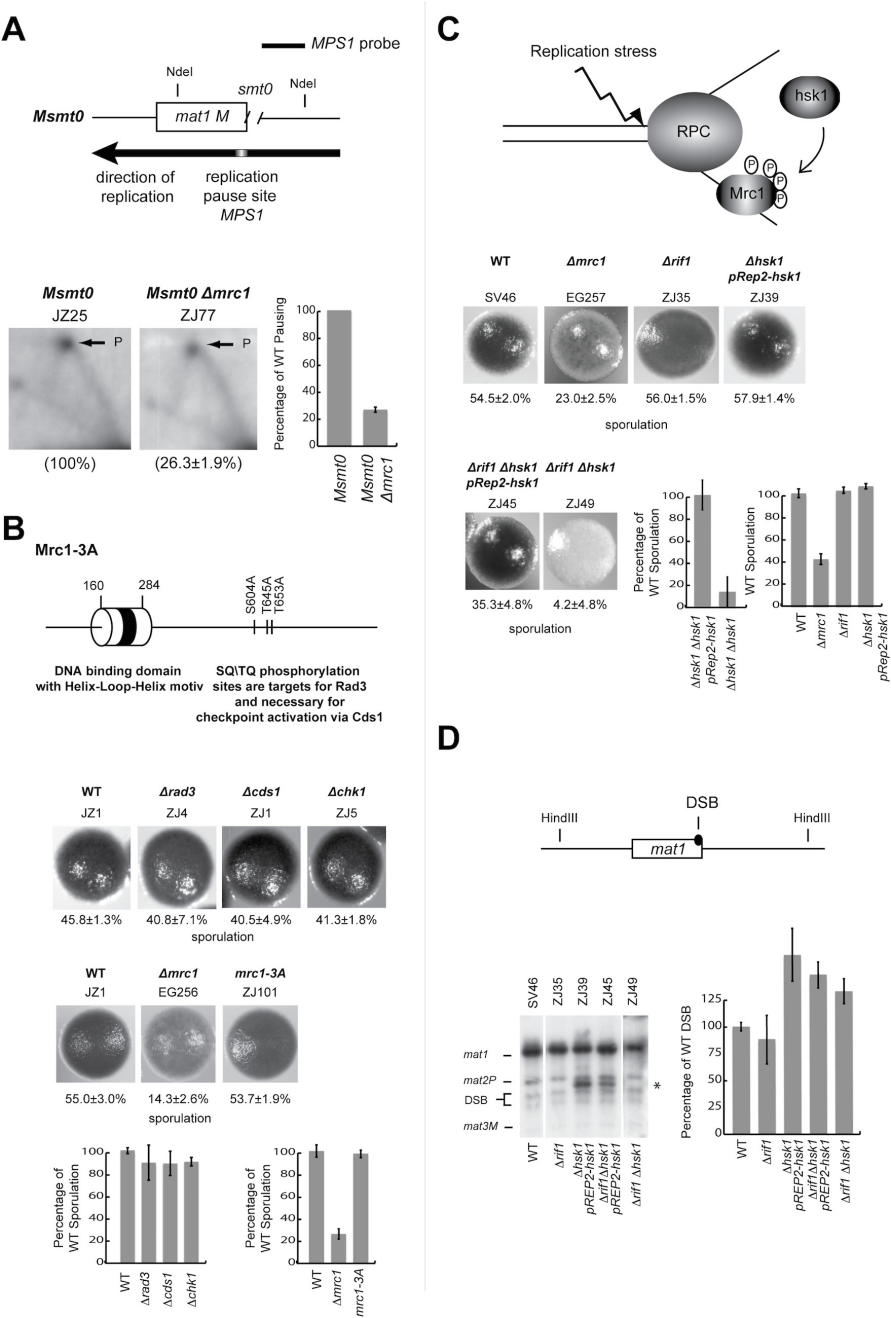
Mrc1 influences pausing at MPS1 independently of the presence and absence of the imprint in *mat1* and its function in the S-phase checkpoint. (A) Quantification of the amount of pausing in an *Msmt0* genetic background. Top panel, line drawing of the *mat1* locus, displaying the position of the *smt0* deletion. Lower panels, 2D-gel analysis of the *mat1* locus from *Msmt0* and *Msmt0 Δmrc1* strains. For a description refer to Fig 2E legend. (B) Effect of the deletion of S-phase checkpoint genes on sporulation. Top line drawing; line drawing; Domain structure of Mrc1. The positions of the DNA binding domain and the Rad3 SQ/TQ phosphorylation sites are indicated. Bottom panel; characterization of the sporulation phenotype of *rad3*, *cds1*, *chk1 and mrc1-3A* mutant strains. Photographs of individual colonies stained with iodine are shown. Below each picture the percentage of sporulation is given. Graphs below show the percentage of sporulation relative to that observed for the wild-type strain (100%). (C) Top line drawing. Replication stress at the replication progression complex (RPC) leads to Hsk1 dependent hyper-phosphorylation of Mrc1. Middle and bottom panels; sporulation phenotypes of given strains. For description see Fig 1C legend. Please note that a decreased sporulation phenotype has previously been observed in a *hsk1-ts* mutant [59]. (D) Quantification of imprinting levels for the strains given above in (C). Asterix indicates signal due to plasmid-probe cross hybridisation. For a description refer to Fig 2B legend.

As outlined in Fig 1A, pausing occurs at the *MPS1* barrier both in imprinted and un-imprinted cells. Since we only observed a reduction in *MPS1* pausing and not a complete loss, an explanation could be that *mrc1* is only required for pausing in one of these two populations. To test if there is a difference in a *Δmrc1* background between the two populations, we analysed the pausing signal in a genetic background where the ribonucleotide imprint was absent due to the *cis*-acting *smt0* deletion [55]. We observed a similar reduction of the pause signal compared to a wild type strain as in the *Δmrc1* strain. (Fig 4A; lower panel). Thus, Mrc1 is required for efficient pausing at the *MPS1* site both in the presence and in the absence of the *mat1* ribonucleotide imprint. This is similar to what has been observed for the other four factors known to be required for pausing at *MPS1* (Lsd1, Lsd2, Swi1 and Swi3) [15,40].

### While the checkpoint function of Mrc1 is not required, the Mrc1 DNA-binding domain is necessary for efficient pausing at *MPS1*

Next we wanted to check whether the observation that Mrc1 regulates efficient replication barrier activity is correlated with one of its known functions. Since Mrc1 is a target for Rad3-dependent phosphorylation as part of the intra-S phase checkpoint (reviewed in Ref. [56]), we first wanted to see whether the S-phase checkpoint plays a role in Mrc1 mediated replication barrier activity. Mrc1 phosphorylation is required for the full activation of the Cds1 effector kinase (reviewed in Ref. [56]). We therefore first tested whether mutation of the *rad3* and *cds1* genes affect sporulation. No such effects were observed (Fig 4B). Similar results have previously been published by Roseaulin *et al*. [57]. We then went on to test whether a checkpoint-inactive allele of *mrc1* (*mrc1-3A* [58]), which carries alanine substitutions of the Rad3 SQ/TQ phosphorylation sites responsible for S-phase checkpoint activation, affected sporulation. Again, no effect was observed (Fig 4B). Finally, a mutation of the Rad3-dependent effector kinase Chk1, which acts in the G2-M checkpoint pathway, did not affect sporulation either (Fig 4B).

Secondly, Mrc1 has been shown to be hyper-phosphorylated by the Hsk1 kinase (*S*. *cerevisiae*, human Cdc7) as a response to replication stress ([59]; Fig 4C). Hsk1 is an essential protein required for initiation of replication, which also has a role in the intra-S phase checkpoint [59,60], but recent work has shown that a double-mutant *ΔrifΔhsk1* is viable [61]. We therefore investigated whether the *Δrif* and *ΔrifΔhsk1* strains displayed decreased sporulation. While the *Δrif* mutation did not affect sporulation, the sporulation level of the *ΔrifΔhsk1* double mutant was strongly reduced (Fig 4C; lower panel). However, when we quantified the levels of imprinting in these strains, we did not detect any decrease in imprinting in the *ΔrifΔhsk1* double-mutant (Fig 4D), showing that the effect of the *hsk1* mutation on sporulation was unrelated to replication pausing and imprinting required for mating-type switching. Thus, *Δhsk1* must affect sporulation in a different manner than that through a role in replication pausing and imprinting.

**Fig 5:**
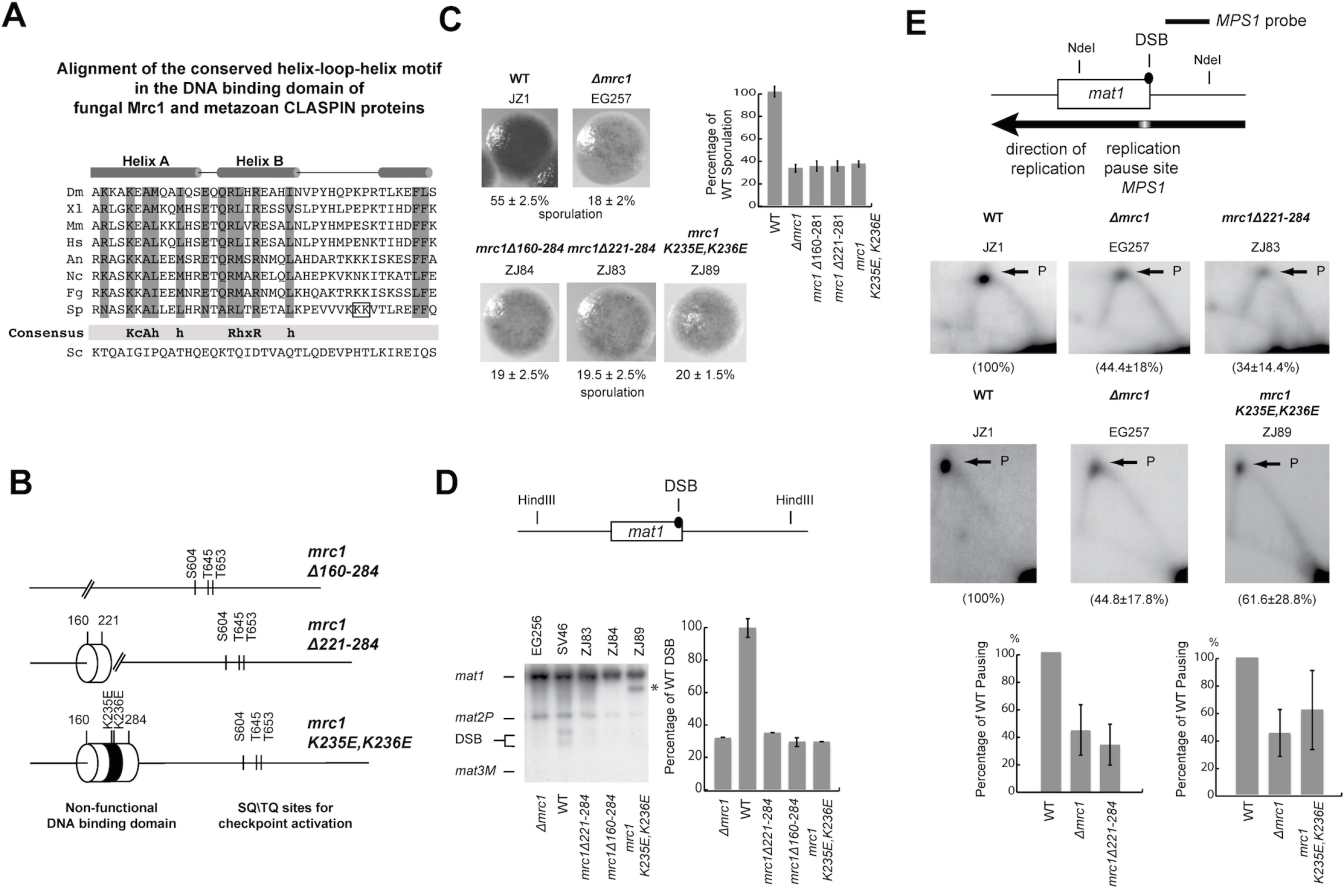
Quantification of the effect of the abolition of the DNA-binding activity of *mrc1* on the *mat1* imprinting and *MPS1* replication pausing. (A) Alignment of the helix-turn-helix DNA binding domain present in the family of Mrc1 proteins. The alignment was made using Clustal [85]. The consensus of the conserved domain is given below. In the last line the corresponding region of *S*. *cerevisiae* Mrc1 is shown. (B) Schematic representation of the mutant *mrc1* alleles. The positions of the mutations affecting the DNA-binding domain and Rad3 phosphorylation sites are given. (C) Left panels; staining phenotypes and sporulation levels of strains carrying the *mrc1* DNA-binding domain mutations (see Fig 1 panel C legend for description). (D) Top line drawing; schematic representation of the mating type region, showing the 10.4 kb HindIII fragment containing the *mat1* locus. Lower left panel, Southern blot of HindIII-digested DNA from *mrc1* strains hybridised with a *mat1P* specific probe. (see Fig 2A & 2B legend for description). Lower right panel, graphical representation of the DSB signal strengths as a percentage of the WT signal’s strength. (E) Top panel; schematic representation of the 2.7 kb NdeI fragment of the mating-type region used to examine replication fork pausing at *MPS1*. Middle panels, 2D-gel analysis of replication pausing at *MPS1* in wild type and mutant strains (for a description see Fig 2 panel B). Lower panel, the replication fork pausing is given for each strain as percentage of the WT pause signal. The mean is given for data obtained from two independent experiments.

In addition to its role in intra-S phase checkpoint activation, *S*. *pombe* Mrc1 also possesses a conserved DNA-binding domain of unknown function ([43], Fig 5A). An alignment of the *S*. *cerevisiae* Mrc1 protein shows that *S*. *cerevisiae* Mrc1 lacks this DNA binding motif (Fig 5A),. Mutation of this helix-turn-helix domain either by deletion or by introduction of two point mutations (Fig 5B) only has a minor effect on the protein’s role in the cellular response to HU treatment [43]. We tested if the mutation of this domain had a similar effect on sporulation, imprinting and replication pausing as the deletion of *mrc1* and this was indeed the case (Fig 5C–5E). Both the domain deletion mutation and the aforementioned two amino acid substitutions reduced the level of sporulation to levels similar to that of the *Δmrc1* mutation. Furthermore, analysis of imprinting levels by quantification of DSB levels at *mat1* confirmed that this result was due to Mrc1’s role in mating-type switching (Fig 5D). Finally, a 2D-gel analysis showed that the *mrc1Δ221–284* and *mrc1-K235E*,*K236E* mutations reduced pausing to similar level as in the *Δmrc1* (Fig 5E).

**Fig 6:**
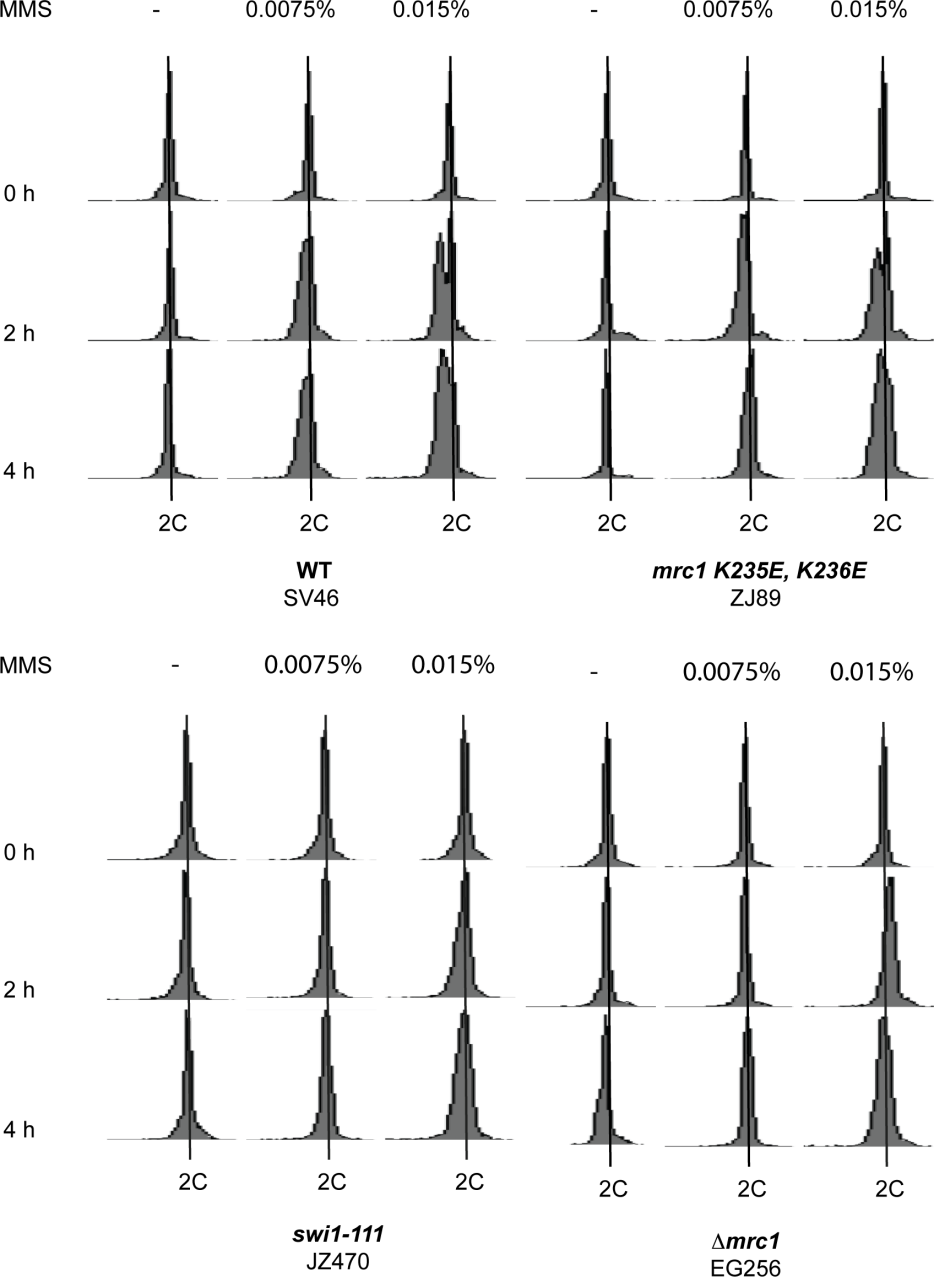
Detection of a functional intra-S phase checkpoint in the *mrc1-K235E*, *K236E* mutant genetic background. WT, *mrc1-K235E*, *K236E*, *Δmrc1*, *swi1-111* strains were grown logarithmic in rich YEA media. Cultures were exposed either to 0%, 0.0075% or 0.015% MMS for 2 and 4 hours. Cells cultures were analysed by FACS as displayed. The concentration of MMS used is given above the panels and the genotypes and strain names below the panels.

Furthermore, we investigated whether the intra-S phase checkpoint is functional in the *mrc1-K235E*,*K236E* genetic background, since the presence of Swi1 and Swi3 at the replication fork is a requirement for this checkpoint. We used a method developed by the Huberman group to test the functionality of the *S*. *pombe* intra-S phase checkpoint [62]. Log-phase *S*. *pombe* cells mainly spent time in the G2 part of the cell cycle and cytokinesis takes place not after mitosis but at the end of the following S-phase. Therefore, cells in G1-,G2/M- and early S-phase all show a 2C DNA content when analysed by FACS. Thus, when log phase cultures are analysed by FACS, we observed only one peak with a 2C DNA content (Fig 6, WT panels “-”MMS). However, if cells with a fully functional intra-S phase checkpoint are exposed to alkylation damage through methyl methanesulfonate (MMS) treatment they will arrest in early S-phase. This allows cytokinesis to take place before S-phase is completed resulting in a peak with a DNA content less than 2C in the FACS analysis (Fig 6, WT 2h & 4h after treatment with 0.015% MMS). This “less than 2C” peak does not appear in cells carrying a mutation leading to defects in the intra-S phase checkpoint (Fig 6, *swi1-111* and *Δmrc1*). When we applied this method to the *mrc1-K235E*,*K236E* mutation strain we observed a wild-type intra-S phase checkpoint response to MMS treatment (Fig 6, *mrc1-K235E*,*K236E* 2h & 4h after treatment with 0.015% MMS).

Our experiments could not detect any correlation between Mrc1’s role in replication pausing and its role in intra-S phase checkpoint activation. Vice versa we did not observe any measurable effect of the *mrc1-K235E*,*K236E* mutations alone on the activation of the intra-S phase checkpoint. However, Zhao et al [43] describe a defect in checkpoint arrest and a “cut” phenotype indicating mis-segregation of genomic DNA, when *Δchk1 mrc1-K235E*,*K236E* double mutant is treated with 12 mM HU (hydroxyurea).

### Loss of Mrc1 reduces the efficiency of pausing

**Fig 7:**
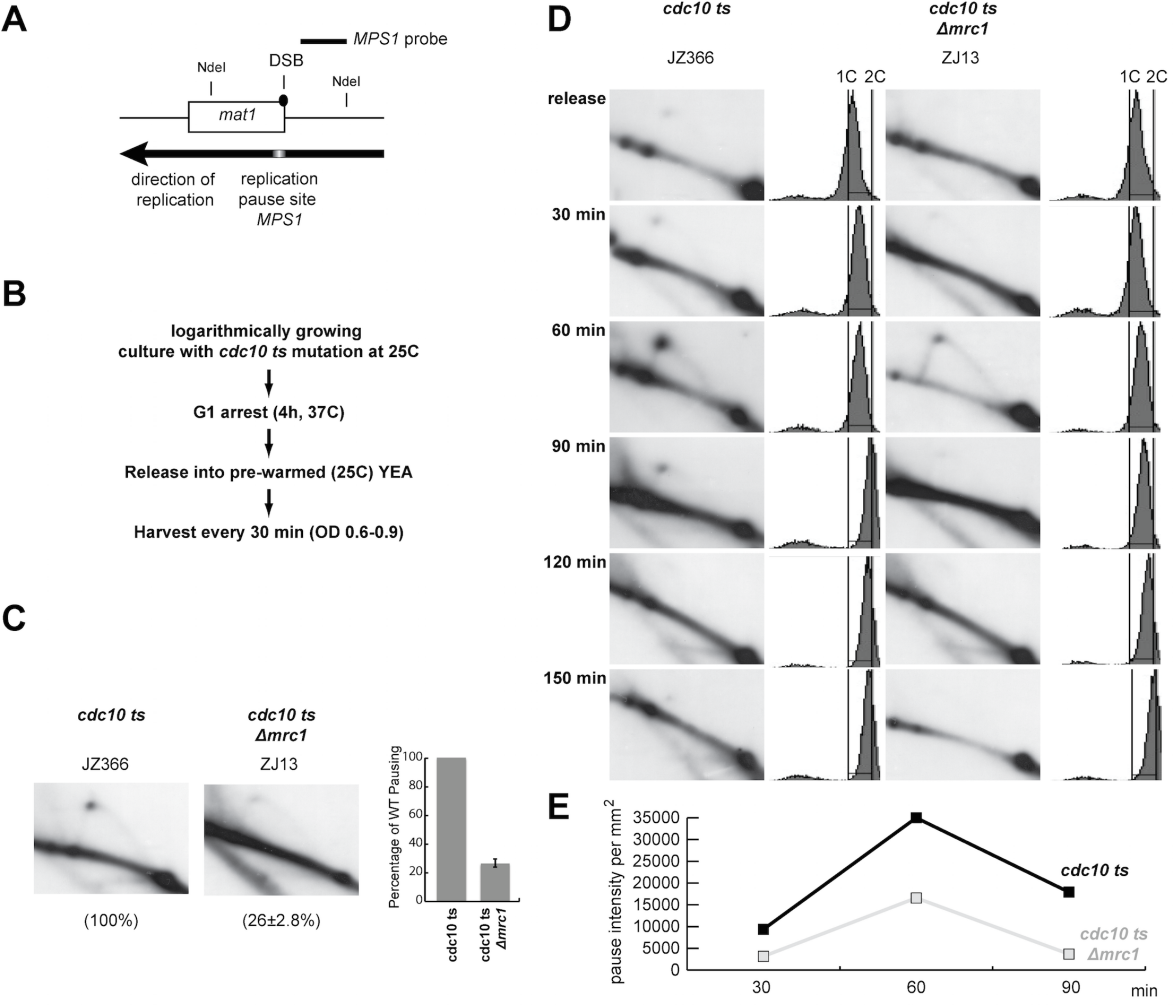
Time-course of replication pausing at the *MPS1* of a *cdc10-ts Δmrc1* mutant and *cdc10-ts* strains. (A) Line-drawing of the analysed region.; Schematic representation of the 2.7 kb NdeI fragment of the mating-type region used to examine replication fork pausing at the *mat1 MPS1*. The position of the DSB and the polarity of replication in this region (black arrow), the replication pause site *MPS1* are shown. (B) Outline of the experimental procedure used for experiment shown in panel D. (C) 2D-gel analysis of log-phase cultures. (D) 2D-gel and FACS analysis of synchronized cultures progressing through S-phase. The analysed region is shown in panel A. Time points are given to the left of the panels. Genotypes and strain names are shown on top of the panels. Experimental procedure is shown in Panel B. (E) Direct quantification of the pause singals’ intensities for experiment shown in panel D. Only the three given time-points were quantified.

Finally we wanted to answer the question whether loss of Mrc1 function leads to a decreased efficiency of replication fork pausing at a barrier or whether the effect we see in an unsynchronized population is due to a decrease in the duration of the pause. To address this we analysed replication intermediates from the *MPS1* pause site (Fig 7A) in synchronized cultures at different time-points after release into S-phase. Compared with WT strains, the number of these intermediates in mutant strains should decrease to the same degree at all time-points if replication forks pause less efficiently. In contrast intermediate numbers should drop more strongly at later time-points if the duration of the pausing is affected. Cultures of *cdc10-ts* and *cdc10-ts Δmrc1* strains were arrested in G1 and released synchronously into S-phase using a program of temperature shifts (Fig 7B). Importantly, a comparison of the *cdc10-ts* and *cdc10-ts Δmrc1* strains detected the same effect of the *mrc1* mutation on *MPS1* pausing for log phase cultures as observed earlier (Fig 7C). This excludes an influence of the *cdc10-ts* mutation on pausing. Furthermore, when synchronized cultures were analysed we observed the same reduction in barrier activity by the *mrc1* mutation at the three time-points a barrier signal was detectable (Fig 7D & 7E). The data suggest that the replication forks are paused with less efficiency and there is no change in the duration of the *MPS1* pause in *Δmrc1* strains. Finally, we would like to note that we observe a slower S-phase progression by FACS analysis in the *Δmrc1 cdc10* strain compared to the *cdc10* strain (Fig 7D), suggesting that loss of *S*. *pombe* Mrc1 affects S-phase progression in a similar manner to what has been observed for *S*. *cerevisiae* (see above).

## Discussion

### All subunits of the heterotrimeric replisome stabilization complex are involved in efficient stalling of the replisome in *S*. *pombe* but not in *S*. *cerevisiae*

In the presented work, we have identified *S*. *pombe* Mrc1 as a novel factor required for efficient replication pausing at the *MPS1* element (Figs 2 and 7). Moreover, we have also shown that *S*. *pombe* Mrc1 is necessary for full barrier activity at the *rDNA* barrier, a *tRNA* gene and at the *RTS1* element (Fig 3). Importantly, these barriers are mediated by different cis-acting DNA-binding proteins including an unknown factor (at *MPS1*), Rtf1 (at *RTS1*), Sap1 and Reb1 (at the *rDNA* barrier), and the polymerase III complex (at the *tRNA* barrier) [26,28,30,48,53]. The seemingly global role at DNA-binding protein-mediated replication barriers reflects what is observed for the other two subunits which form a trimeric replisome sub-complex with Mrc1 (see above), Swi1 and Swi3. But while Swi1 and Swi3 are absolutely required for stalling at the first three barriers [15,16,40], the absence of Mrc1 only reduces barrier activity at these elements about three fold (Figs 2 & 3). This suggests that, unlike Swi1 and Swi3, Mrc1’s role in replisome stalling at barriers is supportive rather than essential, reflecting the different roles of these factors in the DNA replication process (see introduction). The discovery of the involvement of *S*. *pombe* Mrc1 in replication barrier activity is somewhat surprising since previously three laboratories independently have established that *S*. *cerevisiae* Mrc1 is not required for stalling of replication forks at the Fob1 barrier, several tRNA genes and kinetochore binding sites [17,29,38,39]. However, it should be kept in mind that these two organisms are only distantly related [63].

**Fig 8:**
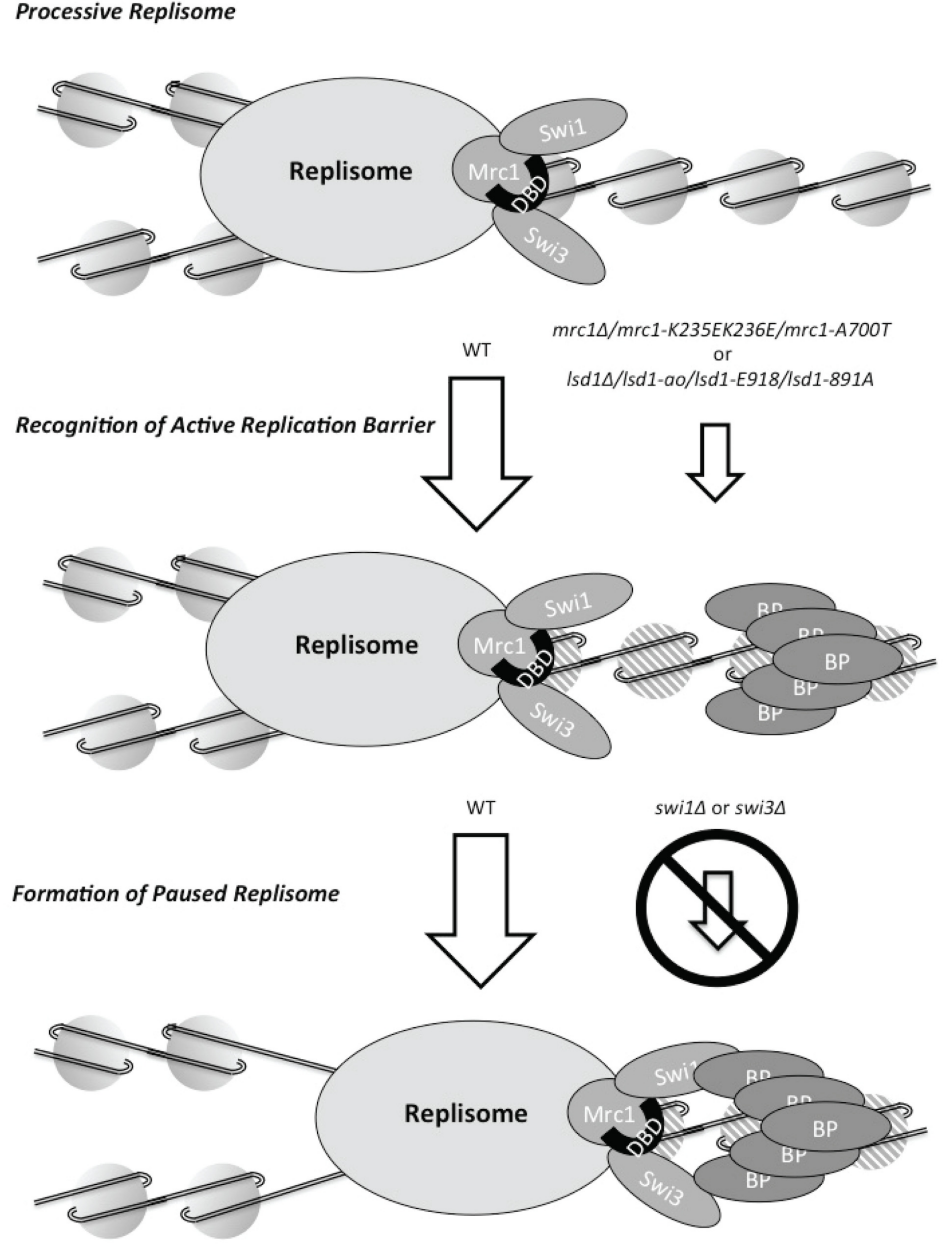
Model of replisome stalling at protein-mediated DNA replication barriers in *S*. *pombe*. The replisome arriving at a DNA replication barrier with a receptive chromatin structure (marked by hatched histones) recognizes the DNA replication barrier by an interaction between the chromosomal DNA and the Mrc1 DNA binding domain. This process facilitates the formation of a stably paused replisome mediated by Swi1, Swi3 and barrier-specific chromatin-bound non-histone proteins (BP). Mutations leading to a loss of function of the catalytic domain of *lsd1* or the *mrc1* DNA binding domain reduce barrier activity. Loss of function mutations of *swi1* or *swi3* abolish barrier activity.

Furthermore, our data suggest a mechanistical explanation for the different role Mrc1 has in replication stalling in the two yeast species. *S*. *pombe* Mrc1 has been shown to posses a helix-turn-helix domain that is phylogenetically conserved in members of this protein family from *S*. *pombe* to human but that is absent from *S*. *cerevisiae* Mrc1 (Fig 5A). This domain has been shown to display an affinity to both double-stranded DNA as well as branched DNA structures [43]. Mutations in the domain only cause a slightly increased sensitivity to HU while the *mrc1* gene-deletion mutant is hypersensitive [43]. Importantly, we demonstrated that loss-of-function point mutations in this Mrc1 DNA-binding domain abolishing DNA binding have the same effect as the complete gene deletion with regards to *mat1* imprinting and *MPS1* pausing (Fig 5). Since Swi1, Swi3 and Mrc1 form a hetero-trimeric complex, one possible explanation for the decrease in replication barrier activity observed in the *mrc1* mutant strains could be that in the absence of a functional Mrc1 DNA binding domain Swi1 and Swi3 are not efficiently loaded onto the replisome. We do not think this explanation is likely. Firstly, because a loss of function mutation in the DNA binding domain does not effect measurably the intra-S phase checkpoint, which is thought to be dependent on Swi1 loading, whereas the checkpoint deficient point mutation *swi1-111* or a complete deletion of *mrc1* abolish the intra-S phase checkpoint (Fig 6). Secondly, it has been shown for *S*. *cerevisiae* that Tof1 and Csm3 do not require Mrc1 to be loaded onto the replisome, although the opposite is true; Mrc1 needs Tof1 and Csm3 for loading [64]. Thirdly, Shimmoto et al. [32] have shown, that Swi3 interacts at WT levels with an Mrc1 fragment, which lacks the DNA binding domain. Vice versa, we have shown that the checkpoint-inactive allele *mrc1-3A* does not cause the sporulation defect associated with a loss of *MPS1* pausing and *mat1* imprinting, showing that it is the DNA binding activity and not the intra-S phase checkpoint activity of Mrc1 that is required at replication barriers (Fig 4). This is further supported by the observation that a deletion of *hsk1*, a gene encoding a kinase which hyperphosphorylates Mrc1 as a response to replication stress [59], does not result in a reduction of *mat1* imprinting (Fig 4). Thus, our discovery suggests that *S*. *pombe* Mrc1 acts to enhance replication stalling at replication barriers by directly interacting with the DNA via its helix-turn-helix domain, a domain absent from *S*. *cerevisiae* Mrc1, rather than via Mrc1’s function in the intra-S phase checkpoint, a function which is conserved for *S*. *cerevisiae* Mrc1(Fig 8). We think it is likely that this function of *S*. *pome* Mrc1 at replication barriers is conserved in other eukaryotes since the DNA binding domain, with the mentioned exception of *S*. *cerevisiae* Mrc1, is conserved among the Mrc1/CLASPIN protein family. Further studies in other model organisms are necessary to better determine if indeed Claspin has a function at replication barriers in higher eukaryotes.

### Mrc1 and its role in replication restart

One of the activities that has been attributed to *S*. *cerevisiae* Mrc1 is its role in replication restart [38]. This is reflected by *Δmrc1* cells decreased ability to re-initiate replication after they have been exposed to HU *[39]*. Since we are looking here at a natural replication pause-site, *MPS1*, where forks are stalled and restarted, we can conclude that *S*. *pombe* Mrc1 is not absolutely required for replication restart. At a natural replication barrier a defect in replication restart would be expected to lead to an increase in the pause signal and the appearance of termination structures, both we do not observe (Fig 2). Nor do we observe slow moving forks as the replication barriers are passed, as previously observed in case of the *rtf2*-mutation at the *RTS1* barrier [42]. Slow moving forks are indicative of a defective or alternative replisome that is restarted after the replication pause. We made similar observations for the *RTS1*, *rDNA* and *tRNA* barriers (Fig 3). However, since we observe residual barrier activity at the barriers analysed, we cannot fully exclude that Mrc1 could both have negative and positive effects on replication re-start processes at all these barriers. Alternative explanations to the absence of an involvement of Mrc1 in replication restart in these experiments, are 1) differences between organisms, (Mrc1 is involved in restart in *S*. *cerevisiae* but not in *S*. *pombe* Mrc1), or 2) differences between restart of replication forks stalled using HU and at natural barriers. Indeed, there is evidence supporting the latter. Firstly, both *S*. *cerevisiae* and *S*. *pombe mrc1* deletion strains are sensitive to transient exposure to HU [43,65]. Secondly, an *mrc1* deletion in *S*. *cerevisiae* did not show significant changes in the intensity of pause or termination signals at the analysed replication barriers [17,29,38,66]. Thirdly, large single-stranded regions have been detected at replication forks stalled in HU, which are thought to be absent or very short at forks stalled at site-specific barriers [67,68]. Fourthly, it has been shown that there is an uncoupling of the replicative helicase from the site of DNA synthesis, when replication forks are stalled using HU in a *Δmrc1* background [18]. In contrast, pausing of the fork does not lead to replisome disassembly and Cdc45, a component of the replicative helicase, could be localised at the site of a paused replication fork in a *S*. *cerevisiae Δmrc1* strain [37]. 2D-gel analysis of replication intermediates from both *S*. *cerevisiae* and *S*. *pombe* cells did not show any evidence for fork collapse at barriers in a *Δmrc1* background ([37], see above). Therefore, so far there is no indication for an uncoupling of replicative helicase and polymerase at replication barriers in a *Δmrc1* background. In conclusion Mrc1 is most likely not required for replication restart at natural replication barriers in both *S*. *cerevisiae* and *S*. *pombe*.

### Two groups of proteins mediate fork arrest in *S*. *pombe*

The observation that some factors only have supportive roles, rather than essential, is not novel, as for example loss of catalytic activity for the lysine–specific demethylases Lsd1/Lsd2 complex only leads to a reduction of *MPS1* activity [40]. It is unknown how Lsd1/Lsd2 is recruited to the replication barriers. So far no interaction between the Swi1/Swi3/Mrc1 and Lsd1/Lsd2 complexes has been demonstrated, and it is not known if there might be a mechanistic link between the functions of these protein complexes. Mutations of Swi1 and Swi3, Mrc1’s interaction partners in the replisome stabilization complex, abolish barrier activity at binding sites of Sap1, Reb1 and Rtf1 completely [24,26–28]. This indicates that there are two groups of proteins, which affect barrier activity in *S*. *pombe* in different ways.

Unfortunately, we do not have enough data to definitively assign roles to the involved proteins or propose a specific mechanism. Nevertheless, it is possible to speculate on the basis of the available data and to propose a tentative hypothesis about the function of the different groups of proteins. Firstly, it is known that Lsd1/Lsd2 complexes regulate transcription of target genes and the position of heterochromatin boundaries (i.e. the accessibility of genomic DNA for transcription factors and other DNA binding proteins) by influencing the methylation status of histones [69–72]. As mentioned above, the activity of replication barriers is reduced when the catalytic activity responsible for normal Lsd1/Lsd2 complex function is lost [40]. It is therefore conceivable, that the chromatin at DNA replication barriers has to be in a receptive state for the incoming replisome to recognize the barrier efficiently (Fig 8). Secondly, we have shown in this study that the presence of a functional Mrc1 DNA binding domain is necessary for the replisome to recognize a DNA replication barrier with optimal efficiency (Figs 5 and 7). An interesting idea would be that an interaction between the replisome and chromosomal DNA at receptive barriers via the Mrc1 DNA binding domain is required for an efficient recognition of the DNA replication barrier (Fig 8). Finally, Swi1 and Swi3 are essential for the formation of a paused replication fork at protein-mediated DNA barriers (see above). Genetic evidence based on the study of the *RTS1* barrier suggest, that they are probably involved in the formation of a stably paused replisome-barrier-complex through the interaction with the staticly bound barrier proteins [15,26] (Fig 8).

### Re-evaluation of the cellular roles of Mrc1

Finally, we suggest that our findings invite to re-evaluate results obtained in earlier studies, which have been using *Δmrc1* strains and contributed observed effects to the loss of checkpoint function of Mrc1. These effects might in fact have been, at least in part, due to loss of Mrc1 DNA-binding and replication barrier activity. For example, in *C*. *elegans*, loss of the *mrc1* homologue leads to some embryonic lethality [73,74]. Previously, these defects have been attributed to Mrc1’s role in checkpoint activation. However, our data raise the possibility that the defects observed are due to Mrc1’s role in replication stalling. Replication barriers could potentially play a role in cellular differentiation and development in higher eukaryotes in a manner similar to fission yeast.

Furthermore, rearrangements of the *rDNA* clusters have been found in a variety of cancer patients with lung and colorectal cancer as well as in cell lines derived from Hodgkin’s lymphoma [75,76]. It has been shown that a deletion of *S*. *pombe mrc1* alone leads to a shortening of the *rDNA* cluster on chromosome III [77]. While this result could be also achieved with a non-phosphorylatable, checkpoint deficient *mrc1-14A* allele, the findings presented in this study raise the question whether this phenomenon also would be observed when the Mrc1 DNA binding activity required for full *rDNA* barrier activity is affected. This question becomes more relevant since a recent study showed that CLASPIN, the human homologue of Mrc1, is stabilised by the deubiquitinating enzyme USP20 during checkpoint activation and that USP20 suppresses xenograft tumor growth [78]. This effect can be initially explained by the influence USP20 has on checkpoint activation. However, a knockdown of TIMELESS (the human counterpart of Swi1/Tof1) has recently been shown not only to cause reduced barrier activity but also an increased collision rate between the transcription and replication machinery in human rDNA repeats [23]. Therefore, it is necessary to revisit the question whether rearrangements in the *rDNA* are only indicators of an increased mutation rate due to a failed checkpoint response or whether they are caused by more local effects due to decreased activity of replication barriers.

## Materials and Methods

### Strains

Strains used in this study are given in S1 Table. Genetic laboratory procedures are given in Ref. [79].

### Genetic screen using Bioneer library

Strain EG9, where the wild-type mating-type region had been tagged with a *LEU2* marker gene, and the version 2 Bioneer knockout library strains (Fig 1B) were grown up in YEA media and mixed in 96 well plates. After mixing, cells were transferred to ELN media using a frogger and allowed to sporulate at 25°C for 5 days. Using the frogger, sporulating cells were transferred to a 2% glucuronidase solution and incubated overnight at 37°C. This step kills cells, and releases the spores from the asci. Spores were washed with water, resuspended in water and transferred to YE plates using the frogger. Germinated colonies were first replica-plated to YEA + 100 *μ*g/ml G418 (selecting for the segregants that carried the gene deletions) + 100 *μ*g/ml cyclohexamide (counter selecting for diploid cells not killed by the glucoronidase treatment) and incubated overnight at 33°C, and then to AA-leu plates (selecting for segregants that carried the wild-type mating-type locus) followed by incubation overnight at 33°C. To asses the sporulation phenotype, strains were replica-plated to PMA+ media followed by incubation at 30°C for three days. Finally, sporulating colonies were stained with iodine vapour.

### Quantification of sporulation

Strains were streaked for single colonies on PMA+ solid media. Plates were incubated at 33°C for two days and then moved to 30°C for one day. The percentage sporulation was determined from at least three different colonies for each strain. The percentage sporulation was calculated as the (number of spores divided by two) divided with the ((number of spores divided by two) plus the number of cells). Average values were calculated. The standard deviation from this averages were displayed as error bars.

### Quantification of imprinting

Strains were grown in 10 ml liquid PMA+ media over night. Cells were spun down, washed with water, re-suspended in 1M Sorbitol and 0.1 M EDTA and treated with zymolase for 2 hours at 37°C. Spheroplasts were spun down and lysed by adding 0.5 ml DNAzol (Invitrogen). The DNA was subsequently precipitated using ethanol, resuspended in 0.5 ml DNAzol and precipitated again. The precipitated DNA was dissolved in 0.5 ml TE, phenol/chloroform extracted and precipitated again using ethanol. The pellet was washed with 70% Ethanol, dried and resuspended in TE. The DNA was digested with HindIII and separated on a 1% agarose gel. The 10.4 kb *mat1P* HindIII fragment was used a probe for the Southern analysis. The intensity of in individual bands was quantified using a phosphor imager. At least two independent measurements were done for each strain. Average values were calculated. The standard deviation from this averages were displayed as error bars.

### Quantification of replication pausing

2D-gel electrophoresis was done as described by Brewer and Fangman [52], except the cells were cultivated in rich YEA media. 50 *μ*g/ml of DNA was digested from each sample. Replication intermediates were purified using BND cellulose [80]. The intermediates were separated in gels containing 0.5% agarose for the first dimension and 1.2% for the second dimension. The intensity of the barrier signals and the ascending part of the Y-arc were quantified using a phosphor imager and corrected for the background signals. Each barrier signals was then normalized by dividing it with the obtained signal from the corresponding ascending Y-arc. To determined the effect of the mutations, we determined the mutant barrier signals intensity relative to the wild-type signal. Importantly, in each experiment the intensity for the mutant and wild-type barrier signals were obtained in parallel such that both mutant and wild-type signals were measured from the same membrane. This is true for the data in all figures except for the time course shown in Fig 7E were the wild-type and mutant signals’ intensities were obtained from separate membranes. Subsequently, both the WT and mutant signal intensities were divided by the WT signal and multiplicated with 100%. At least two independent measurements were taken for each replication barrier analysed. The average intensity of the barrier signals for each strain was calculated and the standard deviation of the data from this average reduction was displayed as error bars. In Fig 7E all time points for each of the two strains were blotted on one membrane and the intensity of the barrier signals subtracted the background signals were directly displayed as a function of time.

### Detection of the intra-S Phase checkpoint

Log-phase cultures, grown in YEA, were exposed to the given concentrations of MMS (Fig 6) [62]. Cells were fixed in 70% ethanol. Flow cytometric analysis was carried out as described in reference [81].

### Time-course experiment

*cdc10*-ts cell cultures were grown overnight at 25°C, diluted and allowed to recover for 1 h at 25°C [82]. The cultures were then incubated at 37°C for 4 h to arrest them with a 1N DNA content. The cultures were released from the block by shifting the temperature to 25°C. Samples were collected for flow cytometry and 2D-gel analysis at 30-min intervals.

## Supporting Information

S1 Table Strainlist. (DOCX)

S2 Table List of deletions causing a severe or complete loss of sporulation. (XLSX)

## References

[1] Dalgaard JZ, Godfrey EL, MacFarlane RJ (2011) Eukaryotic Replication Barriers: How. Why and Where Forks Stall In: Seligmann H, editor. DNA Replication-Current Advances: INTECH pp. 269–304.

[2] Voineagu I, Narayanan V, Lobachev KS, Mirkin SM (2008) Replication stalling at unstable inverted repeats: interplay between DNA hairpins and fork stabilizing proteins. Proc Natl Acad Sci U S A 105: 9936–9941. 10.1073/pnas.0804510105 18632578 PMC2481305

[3] Voineagu I, Surka CF, Shishkin AA, Krasilnikova MM, Mirkin SM (2009) Replisome stalling and stabilization at CGG repeats, which are responsible for chromosomal fragility. Nat Struct Mol Biol 16: 226–228. 10.1038/nsmb.1527 19136957 PMC2837601

[4] Liu G, Myers S, Chen X, Bissler JJ, Sinden RR, Leffak M (2012) Replication fork stalling and checkpoint activation by a PKD1 locus mirror repeat polypurine-polypyrimidine (Pu-Py) tract. J Biol Chem 287: 33412–33423. 22872635 10.1074/jbc.M112.402503 PMC3460443

[5] Krasilnikov AS, Panyutin IG, Samadashwily GM, Cox R, Lazurkin YS, Mirkin SM (1997) Mechanisms of triplex-caused polymerization arrest. Nucleic Acids Res 25: 1339–1346. 9060427 10.1093/nar/25.7.1339 PMC146602

[6] Krasilnikova MM, Mirkin SM (2004) Analysis of triplet repeat replication by two-dimensional gel electrophoresis. Methods Mol Biol 277: 19–28. 15201446 10.1385/1-59259-804-8:019

[7] Krasilnikova MM, Mirkin SM (2004) Replication stalling at Friedreich's ataxia (GAA)n repeats in vivo. Mol Cell Biol 24: 2286–2295. 14993268 10.1128/MCB.24.6.2286-2295.2004 PMC355872

[8] Samadashwily GM, Raca G, Mirkin SM (1997) Trinucleotide repeats affect DNA replication in vivo. Nat Genet 17: 298–304. 9354793 10.1038/ng1197-298

[9] Usdin K, Woodford KJ (1995) CGG repeats associated with DNA instability and chromosome fragility form structures that block DNA synthesis in vitro. Nucleic Acids Res 23: 4202–4209. 7479085 10.1093/nar/23.20.4202 PMC307363

[10] Weaver DT, DePamphilis ML (1984) The role of palindromic and non-palindromic sequences in arresting DNA synthesis in vitro and in vivo. J Mol Biol 180: 961–986. 6098692 10.1016/0022-2836(84)90266-3

[11] Rao BS (1996) Regulation of DNA replication by homopurine/homopyrimidine sequences. Mol Cell Biochem 156: 163–168. 9095473 10.1007/BF00426339

[12] Ahn JS, Osman F, Whitby MC (2005) Replication fork blockage by RTS1 at an ectopic site promotes recombination in fission yeast. EMBO J 24: 2011–2023. 15889146 10.1038/sj.emboj.7600670 PMC1142605

[13] Lambert S, Watson A, Sheedy DM, Martin B, Carr AM (2005) Gross chromosomal rearrangements and elevated recombination at an inducible site-specific replication fork barrier. Cell 121: 689–702. 15935756 10.1016/j.cell.2005.03.022

[14] Dalgaard JZ (2012) Causes and consequences of ribonucleotide incorporation into nuclear DNA. Trends Genet 28: 592–597. 10.1016/j.tig.2012.07.008 22951139

[15] Dalgaard JZ, Klar AJ (2000) swi1 and swi3 perform imprinting, pausing, and termination of DNA replication in S. pombe. Cell 102: 745–751. 11030618 10.1016/s0092-8674(00)00063-5

[16] Krings G, Bastia D (2004) swi1- and swi3-dependent and independent replication fork arrest at the ribosomal DNA of Schizosaccharomyces pombe. Proc Natl Acad Sci U S A 101: 14085–14090. 15371597 10.1073/pnas.0406037101 PMC521093

[17] Mohanty BK, Bairwa NK, Bastia D (2006) The Tof1p-Csm3p protein complex counteracts the Rrm3p helicase to control replication termination of Saccharomyces cerevisiae. Proc Natl Acad Sci U S A 103: 897–902. 16418273 10.1073/pnas.0506540103 PMC1347974

[18] Katou Y, Kanoh Y, Bando M, Noguchi H, Tanaka H, Ashikari T, et al (2003) S-phase checkpoint proteins Tof1 and Mrc1 form a stable replication-pausing complex. Nature 424: 1078–1083. 12944972 10.1038/nature01900

[19] Gambus A, Jones RC, Sanchez-Diaz A, Kanemaki M, van Deursen F, Edmondson RD, et al (2006) GINS maintains association of Cdc45 with MCM in replisome progression complexes at eukaryotic DNA replication forks. Nat Cell Biol 8: 358–366. 16531994 10.1038/ncb1382

[20] Noguchi E, Noguchi C, McDonald WH, Yates JR, Russell P (2004) Swi1 and Swi3 are components of a replication fork protection complex in fission yeast. Mol Cell Biol 24: 8342–8355. 15367656 10.1128/MCB.24.19.8342-8355.2004 PMC516732

[21] Gambus A, van Deursen F, Polychronopoulos D, Foltman M, Jones RC, Edmondson RD, et al (2009) A key role for Ctf4 in coupling the MCM2-7 helicase to DNA polymerase alpha within the eukaryotic replisome. EMBO J 28: 2992–3004. 10.1038/emboj.2009.226 19661920 PMC2760104

[22] Leman AR, Noguchi C, Lee CY, Noguchi E (2010) Human Timeless and Tipin stabilize replication forks and facilitate sister-chromatid cohesion. J Cell Sci 123: 660–670. 10.1242/jcs.057984 20124417 PMC2823575

[23] Akamatsu Y, Kobayashi T (2015) The Human PolI Transcription Terminator Complex Acts as a Replication Fork Barrier that Coordinates the Progress of Replication with rRNA Transcription Activity. Mol Cell Biol. 10.1128/MCB.01521-14 PMC4405639 25776556

[24] Mejia-Ramirez E, Sanchez-Gorostiaga A, Krimer DB, Schvartzman JB, Hernandez P (2005) The mating type switch-activating protein Sap1 Is required for replication fork arrest at the rRNA genes of fission yeast. Mol Cell Biol 25: 8755–8761. 16166653 10.1128/MCB.25.19.8755-8761.2005 PMC1265749

[25] Kobayashi T, Horiuchi T (1996) A yeast gene product, Fob1 protein, required for both replication fork blocking and recombinational hotspot activities. Genes Cells 1: 465–474. 9078378 10.1046/j.1365-2443.1996.d01-256.x

[26] Eydmann T, Sommariva E, Inagawa T, Mian S, Klar AJ, Dalgaard JZ (2008) Rtf1-mediated eukaryotic site-specific replication termination. Genetics 180: 27–39. 10.1534/genetics.108.089243 18723894 PMC2535681

[27] Zaratiegui M, Vaughn MW, Irvine DV, Goto D, Watt S, Bahler J, et al (2011) CENP-B preserves genome integrity at replication forks paused by retrotransposon LTR. Nature 469: 112–115. 10.1038/nature09608 21151105 PMC3057531

[28] Sanchez-Gorostiaga A, Lopez-Estrano C, Krimer DB, Schvartzman JB, Hernandez P (2004) Transcription termination factor reb1p causes two replication fork barriers at its cognate sites in fission yeast ribosomal DNA in vivo. Mol Cell Biol 24: 398–406. 14673172 10.1128/MCB.24.1.398-406.2004 PMC303360

[29] Hodgson B, Calzada A, Labib K (2007) Mrc1 and Tof1 regulate DNA replication forks in different ways during normal S phase. Mol Biol Cell 18: 3894–3902. 17652453 10.1091/mbc.E07-05-0500 PMC1995724

[30] Pryce DW, Ramayah S, Jaendling A, McFarlane RJ (2009) Recombination at DNA replication fork barriers is not universal and is differentially regulated by Swi1. Proc Natl Acad Sci U S A 106: 4770–4775. 10.1073/pnas.0807739106 19273851 PMC2660728

[31] Bando M, Katou Y, Komata M, Tanaka H, Itoh T, Sutani T, et al (2009) Csm3, Tof1, and Mrc1 form a heterotrimeric mediator complex that associates with DNA replication forks. J Biol Chem 284: 34355–34365. 10.1074/jbc.M109.065730 19819872 PMC2797203

[32] Shimmoto M, Matsumoto S, Odagiri Y, Noguchi E, Russell P, Masai H (2009) Interactions between Swi1-Swi3, Mrc1 and S phase kinase, Hsk1 may regulate cellular responses to stalled replication forks in fission yeast. Genes Cells 14: 669–682. 10.1111/j.1365-2443.2009.01300.x 19422421 PMC2837079

[33] Alcasabas AA, Osborn AJ, Bachant J, Hu F, Werler PJ, Bousset K, et al (2001) Mrc1 transduces signals of DNA replication stress to activate Rad53. Nat Cell Biol 3: 958–965. 11715016 10.1038/ncb1101-958

[34] Osborn AJ, Elledge SJ (2003) Mrc1 is a replication fork component whose phosphorylation in response to DNA replication stress activates Rad53. Genes Dev 17: 1755–1767. 12865299 10.1101/gad.1098303 PMC196183

[35] Tanaka K, Russell P (2001) Mrc1 channels the DNA replication arrest signal to checkpoint kinase Cds1. Nat Cell Biol 3: 966–972. 11715017 10.1038/ncb1101-966

[36] Noguchi E, Noguchi C, Du LL, Russell P (2003) Swi1 prevents replication fork collapse and controls checkpoint kinase Cds1. Mol Cell Biol 23: 7861–7874. 14560029 10.1128/MCB.23.21.7861-7874.2003 PMC207622

[37] Calzada A, Hodgson B, Kanemaki M, Bueno A, Labib K (2005) Molecular anatomy and regulation of a stable replisome at a paused eukaryotic DNA replication fork. Genes Dev 19: 1905–1919. 16103218 10.1101/gad.337205 PMC1186190

[38] Szyjka SJ, Viggiani CJ, Aparicio OM (2005) Mrc1 is required for normal progression of replication forks throughout chromatin in S. cerevisiae. Mol Cell 19: 691–697. 16137624 10.1016/j.molcel.2005.06.037

[39] Tourrière H, Versini G, Cordón-Preciado V, Alabert C, Pasero P (2005) Mrc1 and Tof1 promote replication fork progression and recovery independently of Rad53. Mol Cell 19: 699–706. 16137625 10.1016/j.molcel.2005.07.028

[40] Holmes A, Roseaulin L, Schurra C, Waxin H, Lambert S, Zaratiegui M, et al (2012) Lsd1 and lsd2 control programmed replication fork pauses and imprinting in fission yeast. Cell Rep 2: 1513–1520. 10.1016/j.celrep.2012.10.011 23260662 PMC3909218

[41] Ivessa AS, Lenzmeier BA, Bessler JB, Goudsouzian LK, Schnakenberg SL, Zakian VA (2003) The Saccharomyces cerevisiae helicase Rrm3p facilitates replication past nonhistone protein-DNA complexes. Mol Cell 12: 1525–1536. 14690605 10.1016/s1097-2765(03)00456-8

[42] Inagawa T, Yamada-Inagawa T, Eydmann T, Mian IS, Wang TS, Dalgaard JZ (2009) Schizosaccharomyces pombe Rtf2 mediates site-specific replication termination by inhibiting replication restart. Proc Natl Acad Sci U S A 106: 7927–7932. 10.1073/pnas.0812323106 19416828 PMC2683088

[43] Zhao H, Russell P (2004) DNA binding domain in the replication checkpoint protein Mrc1 of Schizosaccharomyces pombe. J Biol Chem 279: 53023–53027. 15471884 10.1074/jbc.M410449200

[44] Egel R, Beach DH, Klar AJ (1984) Genes required for initiation and resolution steps of mating-type switching in fission yeast. Proc Natl Acad Sci U S A 81: 3481–3485. 6587363 10.1073/pnas.81.11.3481 PMC345532

[45] Dalgaard JZ, Klar AJ (1999) Orientation of DNA replication establishes mating-type switching pattern in S. pombe. Nature 400: 181–184. 10408447 10.1038/22139

[46] Vengrova S, Dalgaard JZ (2004) RNase-sensitive DNA modification(s) initiates S. pombe mating-type switching. Genes Dev 18: 794–804. 15059961 10.1101/gad.289404 PMC387419

[47] Vengrova S, Dalgaard JZ (2006) The wild-type Schizosaccharomyces pombe mat1 imprint consists of two ribonucleotides. EMBO Rep 7: 59–65. 16299470 10.1038/sj.embor.7400576 PMC1369229

[48] Sayrac S, Vengrova S, Godfrey EL, Dalgaard JZ (2011) Identification of a novel type of spacer element required for imprinting in fission yeast. PLoS Genet 7: e1001328 10.1371/journal.pgen.1001328 21423720 PMC3053322

[49] Dalgaard JZ, Klar AJ (2001) A DNA replication-arrest site RTS1 regulates imprinting by determining the direction of replication at mat1 in S. pombe. Genes Dev 15: 2060–2068. 11511538 10.1101/gad.200801 PMC312760

[50] Xu H, Boone C, Brown GW (2007) Genetic dissection of parallel sister-chromatid cohesion pathways. Genetics 176: 1417–1429. 17483413 10.1534/genetics.107.072876 PMC1931553

[51] Tanaka T, Yokoyama M, Matsumoto S, Fukatsu R, You Z, Masai H (2010) Fission yeast Swi1-Swi3 complex facilitates DNA binding of Mrc1. J Biol Chem 285: 39609–39622. 10.1074/jbc.M110.173344 20924116 PMC3000942

[52] Brewer BJ, Fangman WL (1988) A replication fork barrier at the 3' end of yeast ribosomal RNA genes. Cell 55: 637–643. 3052854 10.1016/0092-8674(88)90222-x

[53] Krings G, Bastia D (2005) Sap1p binds to Ter1 at the ribosomal DNA of Schizosaccharomyces pombe and causes polar replication fork arrest. J Biol Chem 280: 39135–39142. 16195226 10.1074/jbc.M508996200

[54] Steinacher R, Osman F, Dalgaard JZ, Lorenz A, Whitby MC (2012) The DNA helicase Pfh1 promotes fork merging at replication termination sites to ensure genome stability. Genes Dev 26: 594–602. 10.1101/gad.184663.111 22426535 PMC3315120

[55] Styrkarsdottir U, Egel R, Nielsen O (1993) The smt-0 mutation which abolishes mating-type switching in fission yeast is a deletion. Curr Genet 23: 184–186. 8431959 10.1007/BF00352020

[56] Tanaka K (2010) Multiple functions of the S-phase checkpoint mediator. Biosci Biotechnol Biochem 74: 2367–2373. 21150122 10.1271/bbb.100583

[57] Roseaulin L, Yamada Y, Tsutsui Y, Russell P, Iwasaki H, Arcangioli B (2008) Mus81 is essential for sister chromatid recombination at broken replication forks. EMBO J 27: 1378–1387. 10.1038/emboj.2008.65 18388861 PMC2374842

[58] Yue M, Singh A, Wang Z, Xu YJ (2011) The phosphorylation network for efficient activation of the DNA replication checkpoint in fission yeast. J Biol Chem 286: 22864–22874. 10.1074/jbc.M111.236687 21561865 PMC3123054

[59] Matsumoto S, Shimmoto M, Kakusho N, Yokoyama M, Kanoh Y, Hayano M, et al (2010) Hsk1 kinase and Cdc45 regulate replication stress-induced checkpoint responses in fission yeast. Cell Cycle 9: 4627–4637. 21099360 10.4161/cc.9.23.13937 PMC3166479

[60] Sommariva E, Pellny TK, Karahan N, Kumar S, Huberman JA, Dalgaard JZ (2005) Schizosaccharomyces pombe Swi1, Swi3, and Hsk1 are components of a novel S-phase response pathway to alkylation damage. Mol Cell Biol 25: 2770–2784. 15767681 10.1128/MCB.25.7.2770-2784.2005 PMC1061638

[61] Hayano M, Kanoh Y, Matsumoto S, Renard-Guillet C, Shirahige K, Masai H (2012) Rif1 is a global regulator of timing of replication origin firing in fission yeast. Genes Dev 26: 137–150. 10.1101/gad.178491.111 22279046 PMC3273838

[62] Kumar S, Huberman JA (2004) On the slowing of S phase in response to DNA damage in fission yeast. J Biol Chem 279: 43574–43580. 15297457 10.1074/jbc.M407819200

[63] Forsburg SL, Rhind N (2006) Basic methods for fission yeast. Yeast 23: 173–183. 16498704 10.1002/yea.1347 PMC5074380

[64] Morohashi H, Maculins T, Labib K (2009) The amino-terminal TPR domain of Dia2 tethers SCF(Dia2) to the replisome progression complex. Curr Biol 19: 1943–1949. 10.1016/j.cub.2009.09.062 19913425

[65] Naylor ML, Li JM, Osborn AJ, Elledge SJ (2009) Mrc1 phosphorylation in response to DNA replication stress is required for Mec1 accumulation at the stalled fork. Proc Natl Acad Sci U S A 106: 12765–12770. 10.1073/pnas.0904623106 19515819 PMC2722297

[66] Mohanty BK, Bairwa NK, Bastia D (2009) Contrasting roles of checkpoint proteins as recombination modulators at Fob1-Ter complexes with or without fork arrest. Eukaryot Cell 8: 487–495. 10.1128/EC.00382-08 19234097 PMC2669202

[67] Gruber M, Wellinger RE, Sogo JM (2000) Architecture of the replication fork stalled at the 3' end of yeast ribosomal genes. Mol Cell Biol 20: 5777–5787. 10891513 10.1128/mcb.20.15.5777-5787.2000 PMC86055

[68] Sogo JM, Lopes M, Foiani M (2002) Fork reversal and ssDNA accumulation at stalled replication forks owing to checkpoint defects. Science 297: 599–602. 12142537 10.1126/science.1074023

[69] Lan F, Zaratiegui M, Villen J, Vaughn MW, Verdel A, Huarte M, et al (2007) S. pombe LSD1 homologs regulate heterochromatin propagation and euchromatic gene transcription. Mol Cell 26: 89–101. 17434129 10.1016/j.molcel.2007.02.023

[70] Nicolas E, Lee MG, Hakimi MA, Cam HP, Grewal SI, Shiekhattar R (2006) Fission yeast homologs of human histone H3 lysine 4 demethylase regulate a common set of genes with diverse functions. J Biol Chem 281: 35983–35988. 16990277 10.1074/jbc.M606349200

[71] Gordon M, Holt DG, Panigrahi A, Wilhelm BT, Erdjument-Bromage H, Tempst P, et al (2007) Genome-wide dynamics of SAPHIRE, an essential complex for gene activation and chromatin boundaries. Mol Cell Biol 27: 4058–4069. 17371846 10.1128/MCB.02044-06 PMC1900013

[72] Opel M, Lando D, Bonilla C, Trewick SC, Boukaba A, Walfridsson J, et al (2007) Genome-wide studies of histone demethylation catalysed by the fission yeast homologues of mammalian LSD1. PLoS One 2: e386 17440621 10.1371/journal.pone.0000386 PMC1849891

[73] Ceron J, Rual JF, Chandra A, Dupuy D, Vidal M, van den Heuvel S (2007) Large-scale RNAi screens identify novel genes that interact with the C. elegans retinoblastoma pathway as well as splicing-related components with synMuv B activity. BMC Dev Biol 7: 30 17417969 10.1186/1471-213X-7-30 PMC1863419

[74] Maeda I, Kohara Y, Yamamoto M, Sugimoto A (2001) Large-scale analysis of gene function in Caenorhabditis elegans by high-throughput RNAi. Curr Biol 11: 171–176. 11231151 10.1016/s0960-9822(01)00052-5

[75] MacLeod RA, Spitzer D, Bar-Am I, Sylvester JE, Kaufmann M, Wernich A, et al (2000) Karyotypic dissection of Hodgkin's disease cell lines reveals ectopic subtelomeres and ribosomal DNA at sites of multiple jumping translocations and genomic amplification. Leukemia 14: 1803–1814. 11021756 10.1038/sj.leu.2401894

[76] Stults DM, Killen MW, Williamson EP, Hourigan JS, Vargas HD, Arnold SM, et al (2009) Human rRNA gene clusters are recombinational hotspots in cancer. Cancer Res 69: 9096–9104. 10.1158/0008-5472.CAN-09-2680 19920195

[77] Yasuhira S (2009) Redundant roles of Srs2 helicase and replication checkpoint in survival and rDNA maintenance in Schizosaccharomyces pombe. Mol Genet Genomics 281: 497–509. 10.1007/s00438-009-0426-x 19205745

[78] Zhu M, Zhao H, Liao J, Xu X (2014) HERC2/USP20 coordinates CHK1 activation by modulating CLASPIN stability. Nucleic Acids Res 42: 13074–13081. 10.1093/nar/gku978 25326330 PMC4245974

[79] Moreno S, Klar A, Nurse P (1991) Molecular genetic analysis of fission yeast Schizosaccharomyces pombe. Methods Enzymol 194: 795–823. 2005825 10.1016/0076-6879(91)94059-l

[80] Kiger JA Jr., Sinsheimer RL (1969) Vegetative lambda DNA. IV. Fractionation of replicating lambda DNA on benzoylated-naphthoylated DEAE cellulose. J Mol Biol 40: 467–490. 5364718 10.1016/0022-2836(69)90166-1

[81] Marchetti MA, Kumar S, Hartsuiker E, Maftahi M, Carr AM, Freyer GA, et al (2002) A single unbranched S-phase DNA damage and replication fork blockage checkpoint pathway. Proc Natl Acad Sci U S A 99: 7472–7477. 12032307 10.1073/pnas.112702399 PMC124255

[82] Kim SM, Huberman JA (2001) Regulation of replication timing in fission yeast. EMBO J 20: 6115–6126. 11689451 10.1093/emboj/20.21.6115 PMC125695

[83] Codlin S, Dalgaard JZ (2003) Complex mechanism of site-specific DNA replication termination in fission yeast. EMBO J 22: 3431–3440. 12840005 10.1093/emboj/cdg330 PMC165654

[84] Sanchez JA, Kim SM, Huberman JA (1998) Ribosomal DNA replication in the fission yeast, Schizosaccharomyces pombe. Exp Cell Res 238: 220–230. 9457075 10.1006/excr.1997.3835

[85] Thompson JD, Higgins DG, Gibson TJ (1994) CLUSTAL W: improving the sensitivity of progressive multiple sequence alignment through sequence weighting, position-specific gap penalties and weight matrix choice. Nucleic Acids Res 22: 4673–4680. 7984417 10.1093/nar/22.22.4673 PMC308517

