# Supplementary material for: The DNA-Binding Domain of S. pombe Mrc1 (Claspin) Acts to Enhance Stalling at Replication Barriers
Source: PLoS One. 2015 Jul 22;10(7):e0132595. doi: 10.1371/journal.pone.0132595 (PMC4511789; doi:10.1371/journal.pone.0132595)
Supplement: S1 Table — (DOCX) [file pone.0132595.s001.docx]

***List of strains created for this publication***

EG9 *h90, leu1-32, mat1 Δ1.5kb distal::LEU2, rpl42::cyhR*

EG16 *h90, ade6-216, leu1-32, ura4D-18, mat1 Δ1.5kb distal::LEU2, mrc1-A700T*

EG55 *h90, ade6-216, leu1-32; ura4D-18, mat1 Δ1.5kb distal::LEU2, mrc1-A700T*, w/pMRC1 (=pUR19-mrc1, ZJP004)

EG250 *h90, ade6-210, leu1-32, mat1 Δ1.5kb distal::LEU2, Δmrc1::KanMX6*

EG256 *h90, ade6-210, leu1-32, ura4D-18, Δmrc1::KanMX6*

EG257 *h90, ade6-216, leu1-32, ura4D-18,, Δmrc1::KanMX6*

EG260 *h90, ade6-210, leu1-32, ura4D-18, mrc1-A700T*

JZ1 *h90, ade6-210, leu1-32, ura4D-18*

JZ25 *Msmt0, ade6-210, leu1-32, ura4-D18, his2*

JZ217 *h90*, *ade6-210, leu1-32, ura4-D18,* *mat1 Δ1.5kb distal::LEU2*

JZ366 *h90, ade6-216, leu1-32, cdc10-ts*

JZ470 *h90, ade-210, ura4-D18, swi1-111*

JZ480 *h90, ade-216, swi3-146*

SV46 *h90, ade6-216, leu1-32, ura4D-18*

ZJ1 *h90, ade6-216, leu1-32, ura4D-18, ∆cds1::URA4*

ZJ4 *h90, ade6-216, leu1-32, ura4D-18, ∆rad3::URA4*

ZJ5 *h90, ade6-210, leu1-32, ura4D-18, ∆chk1::URA4*

ZJ13 *h90, ade6-216, leu1-32, Ura4-D18, ∆mrc1::KanMX6, cdc10-ts*

ZJ31 *h90, ade6-210, leu1-32, ura4D-18,* w/pRS113

ZJ32 *h90, ade6-210, leu1-32, ura4D-18, Δmrc1::KanMX6,* w/pRS113

ZJ35 *h90, ade6-210, leu1-32, ura4D-18, ∆rif1::LEU2*

ZJ39 *h90, ade6-210, leu1-32, ura4D-18,* *∆hsk1::KanMX6,* w/pREP2-*hsk1*

ZJ45 *h90, leu1-32, ura4D-18, ∆rif1::LEU2,* *∆hsk1::KanMX6,* w/pREP2-*hsk1*

ZJ49 *h90, leu1-32, ura4D-18, ∆rif1::LEU2,* *∆hsk1::KanMX6*

ZJ59 *h90, ade6-210, leu1-32, ura4-D18, ∆RTS1,* w/pBZ142(RTS1, LEU)

ZJ60 *h90, ade6-210, leu1-32, ura4-D18, ∆RTS1, Δmrc1::KanMX6,* w/pBZ142(RTS1, LEU)

ZJ77 *Msmt0, ade6-216, leu1-32, ura4-D18, Δmrc1::KanMX6*

ZJ83 *h90, leu1-32, mrc1∆221-284*

ZJ84 *h90, leu1-32, mrc1∆160-284*

ZJ89 *h90, ade6-216, leu1-32, mrc1 K235E, K236E*

ZJ98 *h-, leu1-32::pJK148-leu1-pfh1* pfh1D::HphMX4, ura4-D18, his3-D1, arg3-D4 w/pRS113*

ZJ100 *h-, leu1-32::pJK148-leu1-pfh1* pfh1D::HphMX4, ura4-D18, ∆mrc1::KanMX6* w/pRS113

ZJ101 h90, leu1-32, mrc1-S604AT645AT653A::KanR

***List of published plasmids and strains used for this publication or creation of strains***

The following strains and plasmids were used in the construction of the above strains and were very kindly provided by the following colleaques:

pMRC1 pUR19-mrc1, ZJP004, pEG28 isolated from EG55 genomic library transformation

pBZ142 (RTS1, LEU), ZJP009 [1]

pRS113 insert: tRNAGlu08, ZJP011 by M. Whitby [2]

HM532 *h-, leu1-32, ura4-D18, ∆rif1::LEU2, ∆hsk1::KanMX6* w/pREP2-hsk1 by H. Masai [3, 4]

HM533 *h-, leu1-32, ura4-D18, ∆rif1::LEU2, ∆hsk1::KanMX6 w/pREP2-hsk1* by H. Masai [3, 4]

HZ3486 *h-, leu1-32, ura4-D18, mrc1∆160-284* by P. Russell [5]

HZ3488 *h-, leu1-32, ura4-D18, mrc1∆221-284* by P. Russell [5]

HZ3489 *h-, leu1-32, ura4-D18, mrc1 K235E, K236E* by P. Russell [5]

KT1398 *h-, leu1-32, ura4-D18, mrc1 S604A, T645A, T653A-5FLAG-KanMX6*  by Katsunori Tanaka [4]

MS143 *h-, leu1-32, ura4-D18, ∆hsk1::KanMX6,* w/pREP2-hsk1 by H. Masai [3, 4]

MS146 *h-, leu1-32, ura4-D18, ∆mrc1::KanMX6, ∆hsk1::KanMX6* w/pREP2-hsk1 by H. Masai [3, 4]

MS252 *h-, leu1-32, ura4-D18, ∆mrc1::KanMX6* by H. Masai [3, 4]

MS484 *h+, ade6-210, leu1-32, ura4-D18, ∆rif1::LEU2* by H. Masai [3, 4]

MW4907 *h-; leu1-32::pJK148-leu1-pfh1* pfh1D::HphMX4, ura4-D18; his3-D1; arg3-D4* by M.C. Whitby (Steinacher et al 2012 Genes Dev p594) [2]

YM71 *h-, leu1-32, ura4-D18* by H. Masai [3, 4]

***Literature***

1. Dalgaard, J.Z. and A.J. Klar, *A DNA replication-arrest site RTS1 regulates imprinting by determining the direction of replication at mat1 in S. pombe.* Genes Dev, 2001. **15**(16): p. 2060-8.

2. Steinacher, R., et al., *The DNA helicase Pfh1 promotes fork merging at replication termination sites to ensure genome stability.* Genes Dev, 2012. **26**(6): p. 594-602.

3. Hayano, M., et al., *Rif1 is a global regulator of timing of replication origin firing in fission yeast.* Genes Dev, 2012. **26**(2): p. 137-50.

4. Matsumoto, S., et al., *Multiple pathways can bypass the essential role of fission yeast Hsk1 kinase in DNA replication initiation.* J Cell Biol, 2011. **195**(3): p. 387-401.

5. Zhao, H. and P. Russell, *DNA binding domain in the replication checkpoint protein Mrc1 of Schizosaccharomyces pombe.* J Biol Chem, 2004. **279**(51): p. 53023-7.
